# PEGylation Enhances Colloidal Stability and Promotes Ligand-Mediated Targeting of LAF–Xenopeptide mRNA Complexes

**DOI:** 10.3390/polym17222979

**Published:** 2025-11-09

**Authors:** Paul Folda, Eric Weidinger, Johanna Seidl, Mina Yazdi, Jana Pöhmerer, Melina Grau, David P. Minde, Mayar Ali, Ceren Kimna, Ernst Wagner

**Affiliations:** 1 Pharmaceutical Biotechnology, Department of Pharmacy, Ludwig-Maximilians-Universität (LMU) Munich, 81377 Munich, Germany; paul.folda@cup.uni-muenchen.de (P.F.); eric.weidinger@cup.uni-muenchen.de (E.W.); johanna.seidl@cup.uni-muenchen.de (J.S.); mina.yazdi@cup.uni-muenchen.de (M.Y.); jana.poehmerer@cup.uni-muenchen.de (J.P.); melina.grau@cup.uni-muenchen.de (M.G.); 2 Center for NanoScience (CeNS), Ludwig-Maximilians-Universität (LMU) Munich, 80799 Munich, Germany; 3 Institute for Intelligent Biotechnologies (iBIO), Helmholtz Center Munich, 85764 Neuherberg, Germany; david.minde@helmholtz-munich.de (D.P.M.); mayar.ali@helmholtz-munich.de (M.A.)

**Keywords:** colloidal stability, EGFR, mRNA, nucleic acid delivery, PEGylation, polyplexes, targeting, protein corona

## Abstract

For complexation of mRNA into polyplexes, double-pH-responsive lipo-xenopeptides (XP), comprising tetraethylene pentamino succinic acid (Stp) and lipoamino fatty acids (LAFs), were combined with PEGylated lipids, either DMG-PEG 2 kDa (DMG-PEG) or azido-group-containing DSPE-PEG 2 kDa (DSPE-PEG-N3), to increase colloidal stability and to facilitate ligand-mediated targeted mRNA delivery. LAF-XPs mixed with DMG-PEG at low (1.5% and 3%) molar ratios improved colloidal stability and retained transfection efficiency. PEGylation also enabled the formulation of otherwise unstable carrier complexes and prevented aggregation induced by salt, proteins, and serum. PEGylation of more positively charged Stp-LAF_2_ mRNA polyplexes decreased fibrinogen adsorption. More neutral, LAF-rich Stp-LAF_4_ polyplexes exhibited low fibrinogen binding without PEGylation. Intravenous administration of these stabilized mRNA complexes demonstrated enhanced biosafety while preserving transfection efficiency. DSPE-PEG-N3 was selected for cell targeting after strain-promoted azide-alkyne cycloaddition (SPAAC)-mediated click-coupling of DBCO-modified ligands. Higher PEG ratios (10% and 20%) provided effective shielding but reduced transfection efficiency, a drawback known as the “PEG dilemma”. Functionalization with an EGFR-targeting ligand restored transfection in EGFR-positive cell lines in a ligand-specific manner. High transfection efficiency is consistent with a lipophilic-to-hydrophilic polarity switch of LAF-XP carriers upon endosomal protonation, triggering dissociation of the PEG lipids and deshielding of the polyplex.

## 1. Introduction

Therapeutic nucleic acids, such as mRNA, hold great potential for the prevention and treatment of diseases such as cancer and genetic disorders. However, due to their large size and negative charge, nucleic acids are unable to cross the lipid bilayer of cells efficiently. To facilitate this process, a delivery system is required. Viral vectors have proven to be highly effective for this purpose, and the majority of clinical trials rely on them. Their application is limited by restricted cargo capacity, immunogenicity, and challenges in large-scale production [1,2]. Non-viral vectors represent promising alternatives if their efficiencies can be improved. Various carrier systems [3,4,5,6,7,8] have been developed such as lipoplexes [9,10], polyplexes [11,12,13,14,15,16,17], and lipid nanoparticles (LNPs) [18,19]. Among these, LNPs have emerged as the current state-of-the-art delivery system. For instance, Onpattro, an LNP formulation, was the first FDA approved siRNA drug for the treatment of hereditary transthyretin amyloidosis [20]. This was followed by the widely recognized COVID-19 vaccines, Spikevax (Moderna) and Comirnaty (BioNTech) [21,22], both LNP formulations, and also encouraging clinical studies with Cas9 mRNA LNP drug Nexiguran Ziclumeran [23]. LNPs exhibit excellent transfection efficiencies and offer good colloidal stability. However, their formulation process is complex. Typically, they are made of a four-component system containing a helper lipid, cholesterol, a polyethylene glycol (PEG)-conjugated lipid, and an ionizable lipid. While the ionizable lipid complexes nucleic acid and mainly drives transfection, helper lipids are required to generate optimal function. The lipids are soluble only in organic solvents, such as ethanol, while the nucleic acids are dissolved in acidic aqueous buffers. After LNP preparation, the particles must be diluted and dialyzed to remove the organic solvent and adjust the pH to a physiological level.

In contrast, polyplexes and lipoplexes have the advantage of a simple formulation process. Nucleic acid and cationic carrier are mixed in an aqueous buffer, for which there is no need for subsequent dilution or dialysis. Compared to standard four-component LNPs, plain cationic polyplexes and lipoplexes encounter greater challenges in maintaining colloidal stability, making them more prone to aggregation in the presence of salts, proteins, and serum, which can substantially influence their biosafety [16,24]. Furthermore, their higher positive surface charge increases recognition by the innate immune system via complement activation, which potentially triggers inflammation [25,26,27]. Surface modification of nanoparticles with hydrophilic, flexible polymers provided “stealth” properties, i.e., dramatically enhanced colloidal stability and blood circulation times [28]. Shielding polymers such as PEG [29,30,31,32,33,34,35,36,37,38], pHPMA [39,40], poly(2-oxazoline) (pOx) [41,42], and polysarcosine [43,44,45] have been incorporated also into lipoplexes and polyplexes, greatly improving stability and reducing serum–protein interactions of these nucleic acid complexes.

Lipid-conjugated PEG is the key component for maintaining colloidal stability of LNPs [46,47,48,49]. Historically, PEG lipids were first introduced into liposomes and cationic lipid/DNA complexes (lipoplexes) [28,29,50,51,52,53,54,55,56,57]. Two commonly used PEG lipids are DMG-PEG and DSPE-PEG-N3, often applied with a 2 kDa average molecular weight of PEG. Both PEG lipids consist of a hydrophilic PEG polymer linked to a lipid anchor. DMG-PEG contains a shorter anchor composed of two C14 myristic acid chains, whereas DSPE-PEG-N3 features longer C18 stearic acid chains. The shorter lipid anchor of DMG-PEG results in weaker incorporation within the LNP helper lipid surface, which makes it more prone to dissociation (“sheddable PEG”). In the case of the siRNA drug Onpattro and related LNPs, DMG-PEG ensures colloidal stability during formulation and upon intravenous administration, where it detaches from the LNP within the blood stream [58]. This enables apolipoprotein E (apoE) adsorption, which facilitates delivery to hepatocytes. In contrast, PEG lipids with longer hydrocarbon chains, such as DSPE-PEG, are more strongly anchored within the LNP and dissociate more slowly, resulting in longer circulation time [59,60,61,62,63]. Due to this firm anchoring, DSPE-PEG derivatives are also commonly used for cell-specific delivery. The PEG lipids thereby can be functionalized via a reactive terminal linker with a targeting moiety. This enables LNPs to achieve specific delivery via ligand–receptor interactions. Numerous studies demonstrated this approach [64,65,66,67,68,69,70].

**Scheme 1:**
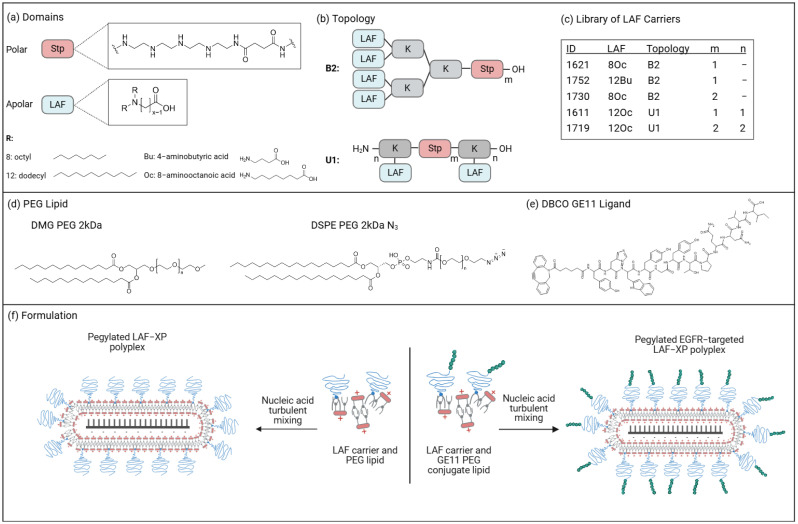
LAF-XP carriers. (**a**) Polar succinoyl tetraethylene pentamine (Stp) domain and apolar lipoamino fatty acid (LAF) domains as structural elements of the carriers. The position of the tertiary amine within the LAF domain can be adjusted by varying the carbon chain length of the ω-amino fatty acids (Bu or Oc) and the length of the N,N-dialkyl substituents (N,N-dioctyl or N,N-didodecyl groups). (**b**) The LAF and Stp domain can be assembled into distinct topologies (B2 and U1) through lysine (K) branching. (**c**) Library of investigated LAF-XP carriers. (**d**) Structures of the two different PEG lipids: polydisperse 1,2-dimyristoyl-rac-glycero-3-methoxypolyethylene glycol-2k (DMG-PEG) and polydisperse 1,2-distearoyl-sn-glycero-3-phosphoethanolamine-N [azido (polyethylene glycol)-2k] (DSPE-PEG-N3) (Average MW 2 kDa, n~45). (**e**) Structure of the DBCO-functionalized GE11 peptide. DBCO-modified GE11 is coupled to DSPE-PEG-N3 via strain-promoted azide-alkyne cycloaddition (SPAAC) to form a DSPE-PEG-GE11 conjugate. (**f**) Formulation of standard PEGylated (**left**) or EGFR-targeted LAF-XP polyplexes (**right**) by adding (rapid pipetting) nucleic acid to LAF carrier/lipid-PEG-GE11 conjugate solution at equal volumes.

Our lab has recently developed double-pH-responsive lipo-amino fatty acid (LAF)-modified xenopeptide carriers (LAF-XPs) that undergo a lipophilic-to-hydrophilic polarity switch upon endosomal protonation [71,72]. LAF-XPs can be synthesized in a precise sequence-controlled manner using fmoc-based solid-phase-assisted peptide synthesis (SPPS). In this process, the polar artificial poly-amino acid succinoyl tetraethylene pentamine (Stp), introduced in properly tBoc protected form, is combined with apolar LAF units via bridging lysine units (Scheme 1a) in various different sequences, ratios, and topologies (Scheme 1b). Formulations with this novel carrier class present a sweet spot between cationic lipoplexes and polyplexes. The carriers share a certain similarity with classical cationic lipids used to form lipoplexes, but instead of a small polar head group they comprise a far larger ionizable polycationic polar domain. With 8 to >10 protonatable nitrogens, they are significantly smaller then commonly applied cationic polymers but display sufficient cationic charges and aqueous solubility to be employed for polyplex formulation [71]. With increased content of LAF units, they also can be used as ionizable lipids to formulate novel LNPs with exceptionally effective endosomal escape properties [73,74]. Formulation of LAF-XP carriers with RNA either as complexes or (in combination with helper lipids and cholesterol) as LNPs has shown exceptional transfection efficiency in vitro and in vivo [71,72,73,74].

The current study aims for the development of LAF-XP mRNA polyplex formulations with increased colloidal stability, offering the potential for shielding against inadvertent unspecific biological interactions and the option for specific cell-receptor-targeted delivery. Noncovalent hydrophobic incorporation of PEG lipid DMG-PEG (for colloidal stabilization and shielding) or DSPE-PEG-N3 (for click-chemistry-based ligand incorporation) into mRNA complexes was considered as fast and feasible strategy. In previously explored lipoplexes and LNPs, PEG lipids are rather stably anchored in the outer lipid shell of the LNP primarily via hydrophobic interactions with helper phospholipids (e.g., DSPC and cholesterol) [75,76]. For the current work we hypothesized that under neutral physiological conditions, the PEG lipids can be successfully anchored within the hydrophobic domain of LAF-XP mRNA complexes but would be released from polyplexes with endosomal protonation of lipidic LAF domains, thus recovering full endosomolytic release capacity.

## 2. Materials and Methods

### 2.1. Materials

Chemically modified CleanCap^®^ FLuc mRNA (5moU) was acquired from Trilink Biotechnologies (San Diego, CA, USA). EZ Cap™ Cy5 Firefly luciferase mRNA (5-moUTP) was bought from Apexbio Technology LLC (Houston, TX, USA). Plasmid pCMVLuc (encoding Photinus pyralis firefly luciferase regulated with a cytomegalovirus promoter and enhancer) was obtained from Plasmid Factory GmbH (Bielefeld, Germany). 1,2-Dimyristoyl-rac-glycero-3-methoxypolyethylene glycol-2000 (DMG-PEG) and 1,2-distearoyl-sn-glycero-3-phosphoethanolamine-N-[carbonyl-azido(polyethylene glycol)-2000] (DSPE-PEG-N3) were bought from Avanti Polar Lipids (Alabaster, AL, USA). Agarose BioReagent low EEO, boric acid, bromophenol blue, ethidium bromide (EtBr) (1% solution in H_2_O), glycerol, RNase-free water, tris(hydroxymethyl) aminomethane hydrochloride (Tris-HCl), and triton X-100 were procured from Sigma-Aldrich (Munich, Germany); 4-(2-hydroxyethyl) 1-piperazineethanesulfonic acid (HEPES) from Biomol (Hamburg, Germany); GelRed (1000×) from VWR (Darmstadt, Germany); and D-(+)-glucose monohydrate, Silver Nitrate (AgNO_3_), disodium carbonate (Na_2_CO_3_), sodium thiosulfate (Na_2_S_2_O_3_), and ethylene diaminetetraacetic acid (EDTA) from Merck (Darmstadt, Germany). The Quant-iT™ RiboGreen RNA Assay Kit was purchased from Thermo Fisher Scientific (Schwerte, Germany) and heparin (5000 I.U. mL^−1^) from B. Braun SE (Melsungen, Germany). All cell culture consumables were purchased from Faust Lab Science (Klettgau, Germany). N2a cells (murine neuroblastoma cell line Neuro2a) and HepG2 cells (human hepatocellular carcinoma) were from the American Type Culture Collection (ATCC, Manassas, VA, USA) and human adherent hepatic carcinoma Huh7 wild-type cell lines from the Japanese Collection of Research Bioresources Cell Bank (Osaka, Japan). The human cervical cancer KB cell line was purchased from the German Collection of Microorganisms and Cell Cultures (DSMZ; Braunschweig, Germany). Low-glucose Dulbecco’s modified Eagle’s medium (DMEM), DMEM Ham’s F12 medium, fetal bovine serum (FBS), penicillin (100 U mL^−1^) and streptomycin (100 µg mL^−1^), trypsin/EDTA, as well as paraformaldehyde (PFA) were bought from Sigma-Aldrich (Munich, Germany) and PAN-Biotech (Aidenbach, Germany). The 5× cell culture lysis buffer and D-luciferin sodium salt were acquired from Promega (Mannheim, Germany. β-mercaptoethanol, 3-(4,5-dimethylthiazol-2-yl)-2,5-diphenyiltetrazolium bromide (MTT), 4′,6-diamidino-2-phenylindole (DAPI), dithiothreitol (DTT), adenosine 5′-triphosphate (ATP) disodium salt trihydrate, coenzyme A trilithium salt, glycylglycine, magnesium chloride (MgCl_2_), and 2,5-DHB and 2-hydroxy-5-methoxybenzoic acid (super-DHB) were acquired from Sigma-Aldrich (Munich, Germany). Polypropylene syringe microreactors were obtained from Multisyntech (Witten, Germany). Trifluoroacetic acid (99%, extra pure) (TFA), sodium hydroxide (1M NaOH), dichloromethane, HPLC reagent grade (DCM), acetonitrile, HPLC reagent grade (ACN), N,N-dimethylformamide (99.8%, extra dry over molecular sieve), croSeal (dry DMF), and acetic acid (CH_3_COOH) were purchased from Thermo Fisher Scientific GmbH (Schwerte, Germany). Sodium dodecyl sulfate (SDS) was purchased from SERVA Electrophoressis GmbH (Heidelberg, Germany). Formaldehyde (CH_2_O) was bought from Grüssig GmbH (Flisum, Germany). Fmoc-protected amino acids; 2-chlorotritylchloride polystyrene resin; dimethylformamide; N-methyl-2-pyrrolidone (NMP); DBCO-NHS; O-(benzotriazol-1-yl)-N; N,N′,N′-tetramethyluronium hexafluorophosphate (HBTU); and piperidine were acquired from Iris Biotech (Marktredwitz, Germany). Diisopropylethylamine (DIPEA), 1-hydroxybenzotriazole hydrate (HOBt), methanol (CH_3_OH), bromophenol blue, and triisopropylsilane (TIS) were purchased from Sigma-Aldrich (Munich, Germany). (Benzotriazol-1-yloxy)-tripyrrolidinophosphonium hexafluorophosphate (PyBOP) was procured from Millipore (Oakville, Canada). Trifluoroacetic acid was obtained from Acros Organics (Geel, Belgium). Linear polyethylenimine (LPEI) 22 kDa was synthesized according to a previously published procedure [77].

### 2.2. Methods

#### 2.2.1. Synthesis of LAF-XP Carriers

The ionizable LAF-Stp carriers were synthesized using solid-phase peptide synthesis (SPPS) according to the protocol reported by Thalmayr et al. [71].

#### 2.2.2. Copper-Free Click Reaction for DSPE-PEG-GE11 Conjugate Formation

Equimolar amounts of DSPE-PEG-N3 and DBCO-GE11/DBCO-GE11scr peptide were mixed and incubated in HBG overnight on a shaker at 25 °C and 250 rpm.

#### 2.2.3. Unmodified and PEGylated LAF-XP Polyplex Formation

The nucleic acid was first diluted in HBG (20 mm of HEPES, 5% (*w*/*v*) glucose, pH 7.4). Separately, the LAF-XP carrier at the specified N/P ratios, along with the selected PEG lipid at defined molar ratios, was diluted in Milli-Q water. For calculating the N/P ratio, all secondary amines within the Stp (succinoyl tetraethylene pentamine) units, the amine groups, and the tertiary amines of the LAFs were taken into account. Equal volumes of nucleic acid solution and LAF carrier/PEG lipid solution were mixed by rapid pipetting and incubated for 40 min at RT in a closed Eppendorf reaction tube. The final concentration of nucleic acid in the LAF-XP polyplex solution was 12.5 μg/mL for mRNA and 10 μg/mL for pDNA, if not otherwise stated. Each carrier was formulated at its respective optimal nitrogen-to-phosphate ratios (N/P): for mRNA, bundles 1621 and 1752 at N/P 24, 1611 at N/P 18, and 1719 at N/P 12, and for pDNA, 1719 and 1730 at a N/P ratio of 12 and 1611 at a N/P ratio of 18.

#### 2.2.4. Zetasizer Measurements

Measurements were carried out using a Zetasizer Nano ZS (Malvern Instruments, Malvern, Worcestershire, UK) using a folded capillary cell (DTS1070) by dynamic and electrophoretic laser light scattering (DLS, ELS). To assess the particle size and polydispersity index (PDI), 40 µL LAF polyplex solutions were prepared as described above (Section 2.2.3), diluted with 40 µL HBG, and analyzed with the following settings: 30 s of equilibration time, temperature 25 °C, refractive index 1.330, and viscosity 0.8872 mPa*s. Each sample was measured three times with six sub-runs per measurement. To determine the zeta potential, the polyplex solution was diluted with 720 µL of HBG and thoroughly mixed by pipetting prior to measurement. Measurement settings were identical to size determination, with the exception of an increase in equilibration time of 60 s. Each sample was measured with 15 sub-runs (10 s each), and zeta potential was calculated by the Smoluchowski equation. All results (size, PDI, and zeta potential) were reported as mean ± SD out of these measurements.

#### 2.2.5. Cell Culture

The human adherent hepatic carcinoma cell lines Huh7 and HepG2 were cultured in Dulbecco’s modified Eagle’s medium (DMEM) supplemented with F12 Ham. KB and N2a (murine adherent neuroblastoma cell line Neuro2a) were cultivated in low-glucose DMEM (1 g/L glucose). All cell culture media were supplemented with 100 U/mL penicillin, 10% fetal bovine serum (FBS), 4 mM of stable glutamine, and 100 µg/mL streptomycin.

#### 2.2.6. Transfections

LAF polyplexes were prepared as described above (Section 2.2.3) for transfections. Cells were seeded 24 h prior to the transfection. For Huh7, 8000 cells/well; for KB, 5000 cells/well; and for N2a and HepG2 cells, 10,000 cells/well were seeded on a 96-well plate. The medium was replaced with fresh medium before transfection. For mRNA, the volumes of 2.0 and 1.25 μL of LAF polyplex solution (12.5 μg/mL mRNA-luc) were added to the corresponding wells in triplicate. In the case of pDNA, 5.0 μL of LAF polyplex solution (10 μg/mL) was added to each well. HBG/H_2_O (50/50) was used as a negative control.

#### 2.2.7. Luciferase Gene Expression Assay

Transfections of LAF polyplexes were carried out as described (Section 2.2.6). After 24 h incubation time, the medium was removed. Next, 100 µL of 0.5× lysis buffer was added to each well, and the cells were stored and frozen overnight at −80 °C. Prior to luciferase expression analysis, plates were brought to RT for 1 h under constant gentle shaking conditions. For mRNA, the cell lysates were diluted 1:100 in PBS. A total of 35 µL of cell lysate was dispensed into an opaque 96-well plate for measurement. Luciferase activity was recorded for 10 s in a Centro LB 960 plate reader luminometer (Berthold Technologies, Bad Wildbad, Germany) after addition of 100 μL LAR buffer (20 mM glycylglycine; 1 mM MgCl2; 0.1 mM ethylenediaminetetraacetic acid; 3.3 mM dithiothreitol; 0.55 mM adenosine 5′-triphosphate; 0.27 mM coenzyme A, pH 8–8.5) supplemented with 5% (*v*/*v*) of a mixture of 10 mM luciferin and 29 mM glycylglycine. Transfection efficiency was calculated as relative light units (RLU) per well. For mRNA, a background (i.e., RLU values of HBG-treated cells) subtraction was performed.

#### 2.2.8. Cellular Uptake Study-Flow Cytometry

At 24 h before the experiment, KB cells were seeded in a 24-well plate at a density of 50,000 cells/well. Transfection was then carried out by applying a volume of 6 µL of LAF polyplexes prepared at a mRNA concentration of 12 µg/mL (20% Cy5-labeled) as described (Section 2.2.3). After 2 h of incubation, the medium was removed, and cells were washed with 400 µL phosphate-buffered saline (PBS). Subsequently 400 µL of heparin was added (1000 IE/mL), and the plate was placed on ice for 10 min to remove polyplexes non-specifically bound to the cell surface. After incubation, heparin was removed, and the cells were again washed with PBS. Then, cells were detached using 100 µL trypsin/EDTA and diluted in 200 µL FACS buffer (PBS supplemented with 10% FBS), supplemented with 0.1% (*v*/*v*) DAPI (1 mg/mL) to stain the nuclei of dead cells. Cellular uptake was quantified using a CytoFLEX S flow cytometer (Beckman Coulter, Brea, CA, USA) with Cy5 excitation at 635 nm and emission detection at 665 nm. Cells were gated based on their forward- and sideward-scatter profiles. At least 14,000–20,000 events were recorded, and data was analyzed by FlowJo 7.6.5 flow cytometric analysis software (FlowJo, Ashland, OR, USA). The results are presented as the mean fluorescence intensity (MFI; *n* = 1) of all live cells.

#### 2.2.9. Steric Stabilization of LAF-XP mRNA Polyplexes Against Salt-Induced Aggregation Through PEGylation

LAF-XP polyplex solutions (40 µL) were formed as described above (Section 2.2.3) and diluted with 70 µL PBS. Subsequently, size was measured as described above (Section 2.2.4).

#### 2.2.10. pH-Triggered Deshielding and Destabilization of LAF-XP mRNA Polyplexes

LAF-XP polyplex solutions (20 µL) were formed as described above (Section 2.2.3) at a concentration of 25 µg/mL. Subsequently, 180 µL of 0.01 mM HCl (pH 4) was added, and the samples were incubated for 30 min at 37 °C under constant shaking conditions (300 rpm). For control groups, 180 µL HBG (pH 7.4) was added, and the samples were incubated under identical conditions. Subsequently, samples were diluted with 700 µL PBS, and the size was measured as described above (Section 2.2.4).

#### 2.2.11. Steric Stabilization of LAF-XP mRNA Polyplexes Against Protein-Induced Aggregation Through PEGylation

Equal volumes of nucleic acid solution and LAF-XP carrier/PEG lipid solution were mixed by rapid pipetting and incubated for 40 min at RT in a closed Eppendorf reaction tube to give 25 µL of LAF-XP polyplex solution with a concentration of 25 μg/mL mRNA. Subsequently, 15 µL of human transferrin solution was added, resulting in a final mRNA concentration of 12.5 µg/mL, with the indicated molar ratios of human transferrin to carrier. After an incubation of 10 min, the PDI and zeta potential were measured as described above (Section 2.2.4).

#### 2.2.12. Serum Assay–Preparation of Serum-Incubated LAF-XP mRNA Polyplexes

LAF-XP polyplexes were formed as described above (Section 2.2.3), using a concentration of 10 µg mRNA/150 µL. The polyplexes were incubated in 90% fetal bovine serum (FBS) for 2 h at 37 °C under continuous shaking conditions at 300 rpm.

#### 2.2.13. DLS Measurements of Serum-Incubated LAF-XP mRNA Polyplexes

For DLS measurements, 40 µL of the FBS-incubated samples was mixed with 40 µL of HBG, yielding a final volume of 80 µL. The solution was transferred to a folded capillary cell. Size was analyzed with the settings described above (Section 2.2.3).

#### 2.2.14. Nanoparticle Tracking Analysis of Serum-Incubated LAF-Xp mRNA Polyplexes

The particle concentration was determined at 25 °C using a NanoSight NS300 (Malvern Instruments, Malvern, UK) equipped with a blue 488 nm laser and sCMOS camera. The FBS-incubated samples (serum dilution 1:10) were diluted 1:200 with HEPES buffer (7.4), resulting in a total dilution of 1:2000. The diluted samples were injected via the integrated syringe pump at a speed of 20 AU following the manufacturer’s instructions. Five runs per sample were conducted. Videos were recorded at a frame rate of 25 fps, with a total of 1498 frames analyzed per measurement. The detection threshold was set to 10. The maximum jump mode, blur, and minimum track length were operated in automatic mode.

#### 2.2.15. Transfection Efficiency Assessment of Serum-Incubated LAF-XP mRNA Polyplexes

Next, 2.25 µL of the serum-incubated LAF polyplexes were added to the corresponding wells in triplicate. As a control, a portion of the original LAF polyplexes was diluted in HBG instead of serum prior to transfection and also added at a volume of 2.25 µL per well in triplicate.

#### 2.2.16. Isolation and Purification of Protein-Corona-Coated LAF-XP mRNA Polyplexes

LAF-XP mRNA polyplexes were formed as described above (Section 2.2.3), using a concentration of 1 µg mRNA/50 µL. The polyplexes were then mixed with mouse serum at a 1:1 volume ratio and incubated for 15 min at 37 °C at 300 rpm. A 0.7 M sucrose solution was prepared by dissolving solid sucrose in Milli-Q H_2_O. The serum-incubated polyplexes (100 µL) were carefully pipetted onto a 300 µL cushion of 0.7 M sucrose and centrifuged at 15,300× *g* for 1 h at 4 °C. Following centrifugation, the supernatant was discarded. The resulting pellet was washed with 400 µL sterile PBS. The pellet was centrifuged again at 15,300× *g* for 5 min at 4 °C, and the supernatant was removed. This washing step was repeated two additional times for a total of three washes. All pipetting steps were performed using sterile filter tips. Subsequently, samples were stored at −20 °C until further analysis (MS analysis and SDS-PAGE).

#### 2.2.17. Protein Corona Determination via Mass Spectrometry (MS) Analysis of Serum-Coated mRNA LAF-XP Polyplexes

Prior to mass spectrometry analysis, samples were prepared as follows: Evotip PURE tips were rinsed with Buffer B (comprising 80% ACN, water, and 0.1% formic acid) and spun down at 800× *g* for 60 s. The tips were then equilibrated in 20 µL of Buffer A (0.1% formic acid) and impulse spun at 800 g for storage until the acidified samples were ready to load. Samples were acidified in 0.4% TFA, and the Evotip PURE was emptied by centrifuging at 800× *g* for 1 min. The acidified samples were loaded onto the Evotip PURE tips and spun at 800× *g* for 1 min. The samples were washed twice with 20 µL of Buffer A and spun down at 800× *g* for 1 min. Elutions were collected in PCR strips by eluting with 20 µL of 45% Buffer B (containing 45% ACN, water, and 0.1% TFA) at 450× *g*. After drying in a SpeedVac and resuspended in 0.1% TFA supplemented with 0.015% DDM, samples were analyzed using liquid chromatography with tandem mass spectrometry (LC−MS/MS; EASY nanoLC 1200, Thermo Fisher Scientific) coupled with a trapped ion mobility spectrometry quadrupole time-of-flight single-cell proteomics mass spectrometer (timsTOF SCP; Bruker Daltonik) via a CaptiveSpray nano-electrospray ion source. A total of 50 ng of sample per injection was loaded on a 5.5 cm High Throughput μPAC Neo HPLC Column (Thermo Fisher Scientific) and analyzed using an 80 min active gradient method at a flow rate of 250 nl min^−1^. Data were analyzed using scanpy (v. 1.10.2) and anndata (v. 0.10.8) in Python 3.11. Thirty independent samples were analyzed from each group (*n* = 2). All proteins expressed in less than half of the samples in each group were filtered out, resulting in 684 proteins used for downstream analyses. The data was log-transformed and normalized per sample. The missing values were input using KNNImputer (n_neighbors = 5) from the sklearn package (v. 1.5.1). With scanpy’s dendrogram function scipy’s hierarchical linkage clustering was calculated on a Pearson correlation matrix over groups for 50 averaged principal components. Differential expression analysis was conducted using Scanpy’s method “rank_genes_groups” with the method set to “*t*-test”. A threshold of *p* < 0.05 and |log fold change| > 1.0 were applied to identify differentially expressed proteins (DEPs). These DEPs were subsequently visualized using volcano plots.

#### 2.2.18. SDS-PAGE and Silver Staining of Protein-Corona-Coated mRNA LAF-XP Polyplexes

Samples were thawed on ice and 20 µL of Milli Q H_2_O, and 10 µL reducing loading buffer (30% glycerol (*v*/*v*); 0.7 mM Tris pH 6.8; 10% SDS (*w*/*v*); 0.12 mg/mL bromophenol blue; 0.93 mg/mL DTT; and 0.01% β-mercaptoethanol (*v*/*v*)) were added. Subsequently, the samples were shaken (300 rpm) at 95 °C for 5 min and applied onto a 3.5–10% gradient SDS gel according to the Laemmli method and separated at 30 mA for 120 min in a Mini-PROTEAN II electrophoresis cell (Bio-Rad, Hercules, CA, USA).

After electrophoresis, the gel was placed in a clean tray and washed three times for 5 min each with 50–100 mL pure H_2_O. The Imperial Protein Stain was gently mixed before use by inverting the bottle several times. Gels were stained for 2 h at room temperature, followed by destaining in ultrapure H_2_O for three days with several water changes until background staining was minimized. Quantification was performed using ImageJ, (version 1.54g). Percentage change (% change) was calculated by using the formula $\%\;change=100\;\times \left(1-\frac{Intensity\;1752}{Intensity\;1611}\right)$ and $\%\;change=100\times \left(1-\frac{Intensity\;PEGylated}{Intensity\;unmodified}\right)$. Three protein bands were quantified at approximately 70, 15, and 8 kDa.

#### 2.2.19. Statistical Analysis

The results are presented as mean values (arithmetic mean) of triplicates. Error bars display the standard deviation (SD). Statistical analysis of the results (mean ± SD) was evaluated by an unpaired *t*-test with Welch’s correction; ns, not significant; * *p* ≤ 0.05, ** *p* ≤ 0.01, *** *p* ≤ 0.001, **** *p* ≤0.0001.

## 3. Results and Discussion

For mRNA delivery, the current best-performing carriers, two U1 U-shape (1611 and 1719) and two B2 bundle LAF-XPs (1621 and 1752), were applied (Scheme 1b,c).

### 3.1. Formulation and Physicochemical and In Vitro Assessment of PEGylated LAF-XP mRNA Polyplexes

LAF-XP polyplexes were assembled in a 50:50 (*v*/*v*) solvent of HBG (HEPES-buffered glucose) and water [78]. To utilize the solvent advantages of LAF-XP polyplexes, DMG-PEG and DSPE-PEG-N3 stock solutions were also prepared in water. This enabled their use in the classic LAF-XP polyplex preparation protocol. A new, slightly modified preparation method was established. Nucleic acid was diluted in HBG. The PEG lipids were co-diluted with the LAF-XP carrier in water at indicated molar ratios of total lipids. Equal volumes of nucleic acid solution and the LAF-XP/PEG lipid solution were mixed by rapid pipetting and incubated for 40 min at RT in a closed Eppendorf reaction tube (Scheme 1f).

To assess the impact of the PEGylation, an in-depth physicochemical characterization, which included dynamic light scattering (DLS), a Ribogreen assay, and an agarose gel shift, was conducted. Subsequently, the PEGylated LAF-XP polyplexes were tested regarding their transfection efficiency and metabolic activity in N2A and KB cell lines. Each carrier was formulated at its respective optimal nitrogen-to-phosphate ratio (N/P) [71,78].

**Figure 1:**
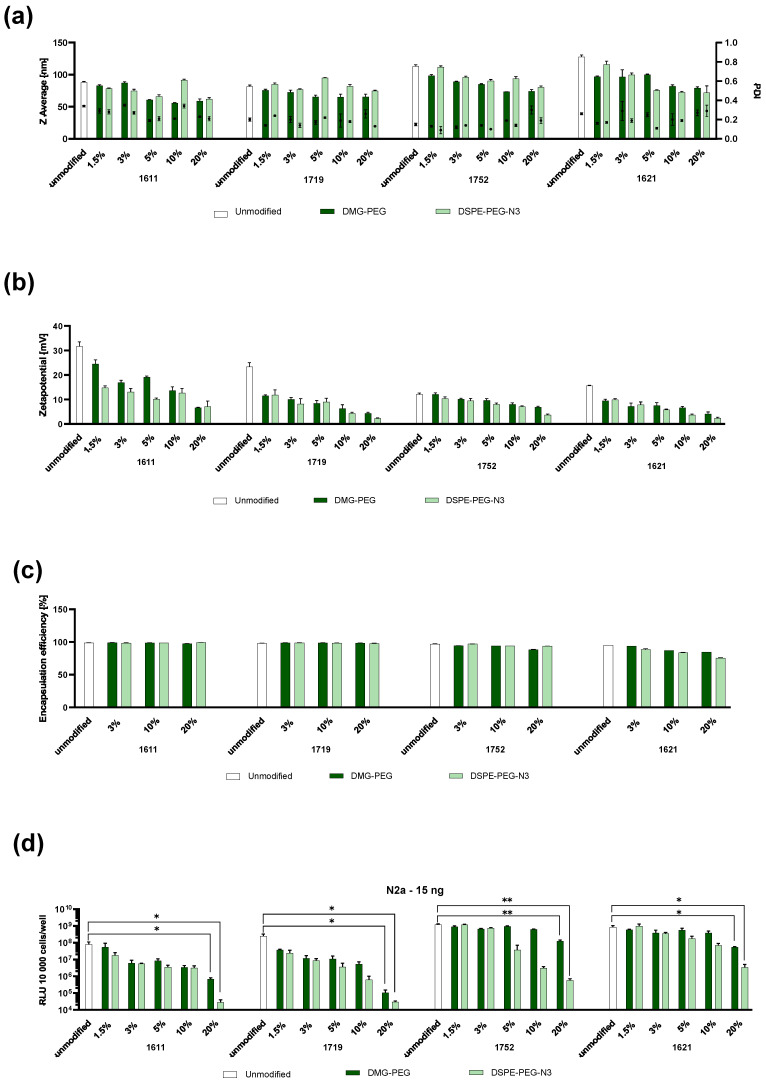
Variation in molar % in PEGylation of LAF-XP mRNA polyplexes with DMG-PEG and DSPE-PEG-N3. (**a**) Hydrodynamic sizes and polydispersity index (PDI) values for unmodified and pegylated (at different molar ratios) LAF polyplexes, measured by dynamic light scattering (DLS) and (**b**) zeta potential analysis determined using electrophoretic light scattering (ELS). (**c**) mRNA encapsulation efficiency at different molar ratios of DMG-PEG and DSPE, determined by the RiboGreen assay. (**d**) Gene transfer activity of unmodified PEGylated LAF polyplexes in N2a cells 24 h after transfection. Comparison of unmodified and PEGylated LAF-XP polyplexes at a dose of 15 ng mRNA-LUC/well. Transfection efficacy was determined by the luciferase gene expression assay (*n* = 3, mean + SD). The statistical significance was determined by an unpaired *t*-test with Welch’s correction; ns, not significant; * *p* ≤ 0.05, ** *p* ≤ 0.01.

For B2 LAF-XP mRNA polyplexes, PEGylation had a particularly favorable effect on the Z-average. Both PEG lipids led to the formation of smaller particles, whereas for U-shapes, only DMG-PEG had a size-reducing effect. In terms of the PDI, no notable changes could be seen (Figure 1a). Regarding the zeta potential, PEGylation led to a reduction for all carriers, and no considerable differences were observed between DMG-PEG and DSPE-PEG-N3 in this regard (Figure 1b).

Previous studies have shown that PEGylation can interfere with nucleic acid compaction [79,80]. Therefore, a RiboGreen assay and a gel shift were conducted. The results indicated that PEGylation did not compromise encapsulation efficiency for the U-shapes 1611 and 1719. However, when incorporated into B2 bundles 1752 and 1621, a reduction in encapsulation, especially at higher molar ratios, could be seen (Figure 1c). These results were confirmed in an agarose gel shift assay. For bundles, PEGylation-induced smearing, particularly at higher molar ratios. For U-shapes, again no notable impact on compaction efficiency could be observed (Figure S3).

**Figure 2:**
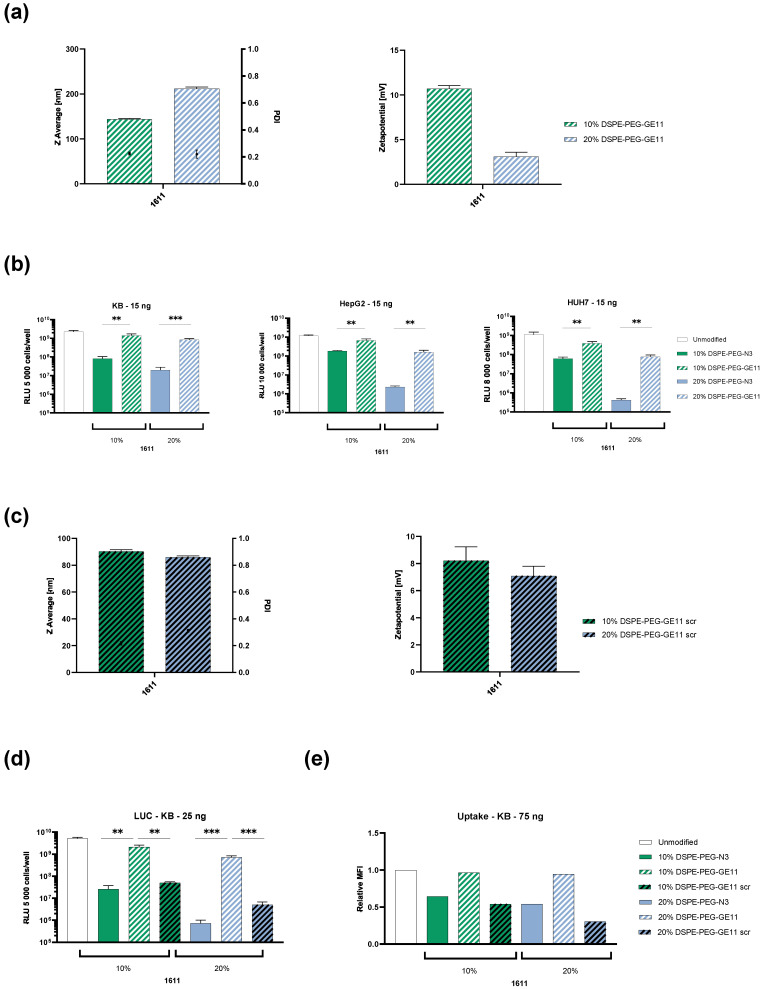
In vitro evaluation of GE11- and GE11scr-functionalized 1611 LAF-XP mRNA polyplexes. (**a**,**c**) Z average, polydispersity index (PDI), and zeta potential of GE11- and GE11scr-functionalized 1611 LAF polyplexes determined by DLS and ELS. (**b**) Comparison of luciferase gene expression of unmodified 1611, PEGylated 1611 (10%/20% DSPE-PEG-N3), and GE11-targeted 1611 (10%/20% DSPE-PEG-GE11) LAF-XP polyplexes on KB, HepG2, and HUH7 cells at a dose of 15 ng mRNA-LUC/well after a total incubation time of 24 h (*n* = 3; mean + SD). (**d**,**e**) Direct comparison of GE11 (EGFR-targeting ligand) and scrambled GE11 (negative control ligand) regarding transfection and uptake efficiency on KB cells. (**d**) Luciferase gene expression was determined after a total incubation time of 24 h at a dose of 25 ng mRNA-LUC/well (*n* = 3; mean + SD). (**e**) Cellular uptake, as determined via flow cytometry (*n* = 1), 2 h after transfection at a dose of 75 ng (20% Cy5-labeled mRNA-LUC) per well (50,000 cells). Data presented as mean fluorescence intensity (MFI) in Cy5-positive cells (Cy5-A) normalized to unmodified LAF-XP polyplexes. The statistical significance was determined by an unpaired *t*-test with Welch’s correction; ns, not significant; ** *p* ≤ 0.01, *** *p* ≤ 0.001.

An increase in PEGylation universally resulted in a decline in transfection efficiency (Figure 2d and Figure S4a). These findings are consistent with the results obtained from DLS measurements, where the zeta potential was decreased by PEG. Previous work has shown that due to the reduced zeta potential, interactions with cellular and endosomal membranes are inhibited. Both cellular uptake and endosomal escape are hindered, which ultimately leads to a reduced transfection efficiency [81,82,83,84,85]. This may also explain why the transfection efficiency of U-shapes was more strongly affected by PEGylation compared to bundles. The decrease in zeta potential was substantially more prominent in U-shapes. In addition, other factors may contribute to the observed differences in behavior. For bundles, PEGylation reduced mRNA compaction, which can often lead to reduced transfection efficiency. However, excessive mRNA compaction can also hinder its release. In the case of bundles, PEGylation may simultaneously lower cellular uptake and endosomal escape while facilitating mRNA release in the cytoplasm. These opposing effects could partially counterbalance each other.

For all carriers, DSPE-PEG-N3 led to a stronger reduction in transfection efficiency than DMG-PEG (Figure 1d and Figure S4a).

DSPE-PEG-N3, featuring a longer lipid anchor (two C18 hydrocarbon chains), likely forms stronger lipophilic interactions with the LAF domain of the carriers. This results in more stable anchoring and long-lasting PEG shielding. In contrast, weaker anchoring of DMG-PEG, due to its shorter lipid anchor (two C14 hydrocarbon chains), allows dissociation from LAF-XP mRNA polyplexes, promoting cell interactions. This likely accounts for the higher transfection efficiency.

This makes DSPE-PEG-N3 suitable for incorporating targeting ligands. Its azido group enables functionalization with targeting moieties via copper-free azido/DBCO click chemistry.

Furthermore, an MTT assay revealed no substantial effect of PEGylation on metabolic activity (Figure S4b,c).

**Scheme 2:**
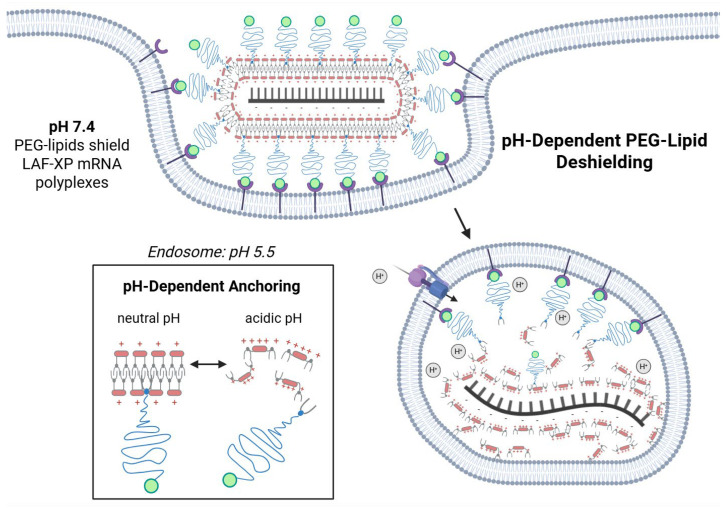
Hypothetical model of receptor-targeted delivery and endosomal deshielding. At physiological pH, the PEG lipids are well anchored within the hydrophobic LAF domains of the carriers and shield the LAF-XP mRNA polyplexes. Cell membrane interactions and subsequent uptake occur predominantly through ligand–receptor interactions. Endosomal acidification leads to the protonation of the LAF domain, resulting in a polarity shift, solubilization of the nanoparticle and dissociation of PEG lipids from the polyplex. As a result, interactions of the LAF carriers with the endosomal membrane are restored, enhancing endosomal escape.

Finally, the anchoring mechanism of the PEG lipids under physiological and endosomal acidic conditions was evaluated (Figure S5). The size and zeta potential of unmodified and fully shielded (20% DSPE-PEG-N3) LAF-XP mRNA polyplexes were compared after an incubation at either pH 7.4 or at pH 5.4. As observed before, under physiological conditions the shielded particles exhibited a strongly reduced zeta potential. Upon acidification, however, the zeta potential experienced a sharp increase (9.4-fold for 1611, 3.8-fold for 1621), suggesting a loss in shielding. These findings support the hypothesis that the PEG lipids are released from polyplexes with endosomal protonation of the lipidic LAF domains (Scheme 2). For the unmodified formulations, the rise in zeta potential (1.4 for 1611 and 1.9 for 1621) was not as drastic as for the shielded ones.

### 3.2. Targeting of LAF-XP mRNA Polyplexes with GE11

Tumor-specific nucleic acid delivery still remains challenging [86]. The particles must be shielded, meaning they should exhibit a neutral zeta potential to prevent nonspecific cellular interactions. To ensure delivery to the intended site, a targeting moiety has to be incorporated. A promising approach involves the use of ligands, which have demonstrated high potential in various studies [32,87,88,89,90,91,92,93,94]. For instance, GE11 is a phage-derived peptide ligand and consists of 12 amino acids with the sequence H_2_N-YHWYGYTPQNVI-COOH, specifically targeting the epidermal growth factor receptor (EGFR). It has shown encouraging results both in vitro and in vivo [95,96,97,98,99]. In the following study (Scheme 2), the GE11 peptide was functionalized with an N-terminal DBCO moiety. This enables azido/DBCO copper-free click reactions to DSPE-PEG-N3, yielding a DSPE-PEG-2k-GE11 conjugate (DSPE-PEG-GE11). The identity and purity of the DBCO-GE11 peptide conjugate was confirmed by MALDI-TOF-MS and HPLC (Figure S1). For mRNA, DSPE-PEG-N3 at molar ratios of 10% and 20% exhibited a significantly decreased zeta potential and transfection efficiency for all carriers. These molar ratios are ideal for targeting. Consequently, 1611 was selected for targeting studies due to its superior mRNA compaction at high molar ratios of PEG. In accordance with the newly established formulation protocol, the DSPE-PEG 2 kDa-GE11 conjugate was used in the same manner as DSPE-PEG-N3 (Scheme 1f).

DLS measurements revealed that GE11-functionalized mRNA particles exhibited small, uniform sizes with a neutral zeta potential (Figure 2a).

Three EGFR-expressing cell lines HEPG2, HUH7, and KB were selected to assess the transfection efficiency of GE11-functionalized 1611 LAF-XP polyplexes. Unmodified, shielded (10% and 20% DSPE-PEG-N3), and targeted (10% and 20% DSPE-PEG-GE11) formulations were evaluated. Shielded 1611 exhibited lower transfection efficiency, which was restored by GE11 functionalization (Figure 2b).

In order to investigate the specificity of the ligand–receptor interaction, a constitutional isomer of GE11 (GE11-scr) was synthesized (Figure S2). This was first reported by Yu et al.; in their study, the original amino acid sequence (H_2_N-YHWYGYTPQNVI-COOH) was scrambled, resulting in a new sequence: H_2_N-YWGPNIHYYTQV-COOH [100]. In the same way as for the GE11 peptide, the GE11-scr peptide was functionalized with a DBCO group and conjugated with DSPE-PEG-N3.

GE11-scr-functionalized 1611 LAF-XP polyplexes formed smaller particles with a higher zeta potential compared to those functionalized with GE11 (Figure 2a,c). However, unlike GE11, the scrambled GE11 did not enhance transfection efficiency. No considerable differences were observed between shielded and GE11-scr-functionalized particles (Figure 2d). Subsequent uptake measurements supported these findings. Shielding reduced cellular uptake, while GE11-functionalized LAF-XP polyplexes recovered uptake nearly to the extent of unmodified LAF-XP polyplexes. In contrast, GE11-scr-functionalized LAF-XP polyplexes exhibited uptake levels similar to shielded particles (Figure 2e).

### 3.3. PEGylation and GE11 Targeting of LAF-XP pDNA Polyplexes

For analogue pDNA investigations, U1 U-shapes 1611 and 1719, as well as the B2 bundle 1730, were selected (Scheme 1b,c). Similar trends to those for mRNA were observed. DLS measurements revealed that the size and PDI remained largely unchanged upon PEGylation, whereas zeta potential was reduced (Figure S6a,b). Furthermore, an ethidium bromide (EtBr) assay showed no substantial impact of PEGylation on pDNA binding ability (Figure S6e). LAF–XP pDNA polyplexes are generally more stable than their mRNA counterparts. They tend to aggregate less during particle formation, can be prepared at lower N/P ratios, and compact pDNA better than mRNA. These features likely explain why PEGylation has little impact on pDNA compaction, whereas it can affect the compaction of mRNA polyplexes.

No considerable differences were detected between DMG-PEG and DSPE-PEG-N3 in the physicochemical evaluation. However, regarding transfection efficiency, DSPE-PEG-N3 led to a stronger reduction than DMG-PEG (Figure S6c). As observed with LAF-XP mRNA polyplexes, this can again be attributed to the differing anchor lengths of the two different PEG lipids.

For GE11 targeting, unmodified and shielded (25% DSPE-PEG-N3) 1719 was compared to targeted (25% DSPE-PEG-GE11) 1719. Shielding resulted in a lower transfection efficiency, confirming the results from the initial screening. GE11 functionalization led to the recovery of the transfection efficiency in HepG2 cells, and in HUH7 cells it even outperformed the unmodified formulation (Figure S7b).

### 3.4. Colloidal Stability

In vitro evaluation revealed that DMG-PEG at low molar ratios does not considerably reduce transfection efficiency. Previous work has shown that the addition of small amounts of PEG also improves the colloidal stability of cationic polymer delivery systems [35,101,102]. In subsequent experiments, we aim to improve colloidal stability with low amounts of DMG-PEG but not at the cost of reduced transfection efficiency.

#### 3.4.1. Steric Stabilization of LAF-XP mRNA Polyplexes Against Salt-Induced Aggregation Through PEGylation

The ionic strength of the physiological environment can present a substantial risk to the stability of cationic delivery systems. Salts can interfere with the electrostatic interactions between the cationic carrier and the anionic nucleotide backbone, leading to aggregation [16,103]. PEG on the surface of polyplexes can provide protection against salt-induced aggregation through steric stabilization [32,104,105,106,107,108,109]. In a similar manner, we investigated whether PEGylation could sterically stabilize the LAF-XP polyplexes. We chose two molar ratios of DMG-PEG (3% and 10%) and compared them to unmodified carriers. After LAF-XP polyplex formation, an aliquot of phosphate-buffered saline (PBS) was added, and size was determined at different time points.

**Figure 3:**
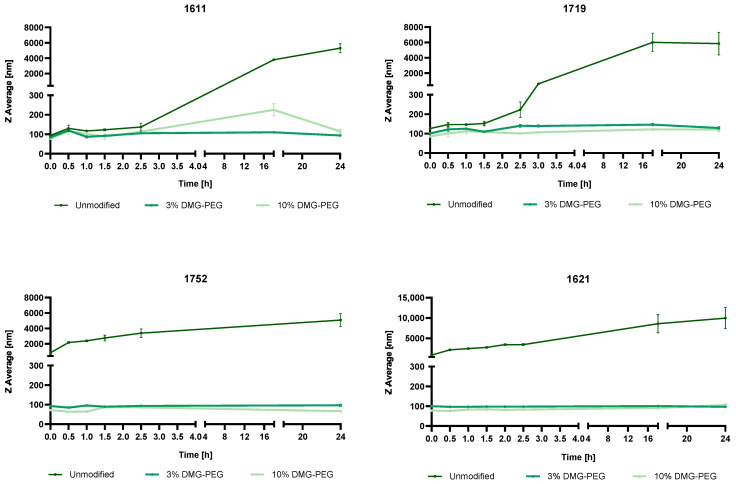
Stabilization of LAF-XP mRNA polyplexes against salt-induced aggregation via PEGylation with DMG-PEG. Kinetic study of the hydrodynamic size of unmodified and PEGylated (3% and 10% DMG-PEG) LAF-XP polyplexes in the presence of PBS. Following LAF-XP polyplex formulation, PBS was added, and the particle size was monitored at the indicated time points via dynamic light scattering (DLS).

Interestingly, unmodified LAF-XP mRNA polyplexes exhibited topology-dependent differences in colloidal stability upon PBS addition. B2 bundles (1752 and 1621) aggregated immediately. U-shapes were more stable, with 1719 aggregating after 150 min and 1611 only after 18 h (Figure 3). The difference in stability is likely due to the different structural and cationic properties of B2 bundles and U-shapes. The B2 bundles are more hydrophobic and less cationic due to a higher content of LAF residues that are not protonated at physiological pH. In contrast, U-shapes have a lower LAF content and include an additional primary amine in their backbone. This presumably enables stronger electrostatic interactions and compaction of mRNA. A stronger binding in U-shapes is consistent with delayed salt-induced aggregation. PEGylation with both 3% and 10% DMG-PEG prevented aggregation completely in bundles and U-shapes, even after 24 h (Figure 3). Especially, the B2 bundles, with weaker binding, therefore benefit more from PEGylation.

The strong increase in zeta potential under endosomal conditions as observed in Section 3.1 suggests that protonation of the LAF carriers leads to dissociation of the PEG lipids from the polyplexes. Consequently, a functional loss of stability would also be predicted in the presence of salt. To verify this, a second PBS stability study was conducted with 1621 polyplexes (Figure S8). Unmodified and PEGylated polyplexes (0.5% and 1% DMG-PEG) were incubated under acidic conditions before PBS was added for incubation in salt at neutral pH. As a control, standard PEGylated polyplexes (without acidic preincubation) were incubated at pH 7.4. Consistent with the previous findings, unmodified 1621 LAF-XP mRNA polyplexes were immediately aggregated with PBS, whereas PEGylated polyplexes remained stable upon incubation with PBS at pH 7.4. In contrast, PEGylated polyplexes pre-exposed to acidic conditions exhibited aggregation in PBS pH 7.4. The deshielded PEGylated polyplexes aggregated at a markedly slower rate than their unmodified counterpart (Figure S9). This suggests that PEG lipids are only partially released under acidic conditions. Nevertheless, this partial deshielding is apparently sufficient for restored high transfection efficiency (Scheme 2 and Figure 2).

#### 3.4.2. Reduction in the N/P Ratio Through PEGylation

Bundles 1621 and 1752 require a higher N/P ratio (N/P 24) to form small and stable particles. At lower N/P ratios, these carriers tend to aggregate, especially at higher concentrations. We investigated whether the bundles 1621 and 1752 could be formulated at half of the standard N/P ratio (N/P 12 instead of N/P 24) by incorporation of low molar amounts of DMG-PEG (1.5% and 3%) at in vivo concentration 33.3 µg/mL (5 µg mRNA in 150 µL).

As expected, unmodified 1752 and 1621 aggregated at N/P 12. The incorporation of low molar amounts of DMG-PEG led to the formation of small and stable particles with low PDIs and successfully prevented aggregation (Figure S9).

#### 3.4.3. Overcoming mRNA LAF-XP mRNA Polyplex Instability Through PEGylation

**Figure 4:**
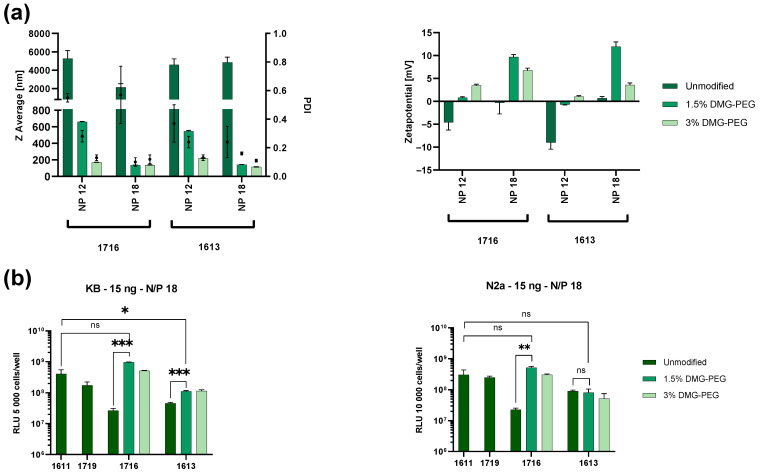
Stabilization of LAF-XP mRNA polyplexes with aggregation-prone carriers 1613 and 1713 and reduction in N/P ratio. (**a**) Z average, polydispersity index (PDI), and zeta potential of unmodified and PEGylated (1.5% and 3% DMG-PEG) 1716 and 1613 polyplexes at N/P ratios of 12 and 18, determined by dynamic light scattering (DLS) and electrophoretic light scattering (ELS). (**b**) The transfection efficiency of unmodified, aggregation-prone 1716 and 1613 carriers was compared to that of their stabilized PEGylated counterparts (1.5% and 3% DMG-PEG), at N/P 18. The 1611 (N/P 18) and 1719 (N/P 12) polyplexes were included as a control group and represent the current gold standard for mRNA transfection in the U-shape class. Luciferase gene expression was measured at 15 ng mRNA-LUC/well (*n* = 3, mean ± SD). The statistical significance was determined by an unpaired *t*-test with Welch’s correction; ns, not significant; * *p* ≤ 0.05, ** *p* ≤ 0.01, *** *p* ≤ 0.001.

Encouraged by the successful reduction in the N/P ratio in 1752 and 1621, we hypothesized that the PEGylation strategy can be applied to carriers that previously could not be formulated. Thalmayr et al. reported that carriers 1716 and 1613 failed to form stable particles due to severe aggregation [71]. Structurally, 1716 and 1613 are highly similar. Both contain 12Oc as their LAF unit and have nearly identical molecular weights. They only differ in their topology. Carrier 1613 is a B2 bundle, while 1716 is a U4 U-shape. The combination of four LAF 12Oc motifs with a single Stp unit appears to result in highly unstable structures. However, both carriers were successfully formulated as LNPs, with 1716 demonstrating a particularly high transfection efficiency [74]. The carriers were PEGylated (1.5% and 3% DMG-PEG) at the N/P ratios of 12 and 18 (Figure 4a). At N/P 12, both unmodified carriers underwent aggregation. However, PEGylation significantly improved size and uniformity. It was found that 1.5% DMG-PEG successfully prevented aggregation, although it resulted in larger particles. Nevertheless, the formulations exhibited good uniformity with favorable PDIs. In addition, 3% DMG-PEG facilitated optimal particle formation for both 1613 and 1716. Notably, these formulations exhibited very low zeta potential, approaching near-neutral values. At N/P 18, both 1.5% and 3% DMG-PEG modification resulted in the formation of small particles with a low PDI and positive zeta potential.

Subsequently, the different formulations were tested regarding their transfection efficiency in N2a and KB cells. They were compared to 1611 and 1719, which represent the current gold standard for mRNA transfection within the U-shape class. Carrier 1716 with 1.5% DMG-PEG outperformed both unmodified 1611 and 1719 in transfection. In contrast, unmodified 1716 aggregates exhibited poor transfection efficiency, emphasizing the positive impact of PEGylation. Carrier 1613 demonstrated limited transfection efficiency compared to the other carriers. Here, PEGylation only had a positive effect on transfection efficiency in KB cells, where a slight increase in transfection efficiency was observed (Figure 4b).

Our previous work demonstrated that 1719 had a lower transfection efficiency in vivo compared to 1611, 1621, and 1752 [71]. Therefore, the focus in subsequent experiments was placed on these three in vivo best-performing carriers.

### 3.5. Protein Corona

In vitro transfection activity often does not match in vivo activity [110,111,112]. Upon exposure of nanoparticles to biological fluids, a protein corona forms on the surface that strongly influences further distribution and biological efficacy [113,114,115,116,117,118,119,120,121,122,123,124,125]. The novel LAF-XP mRNA polyplexes exhibit unexpectedly high transfection efficiency in the presence of full serum even at a very low dose [71,72]. This is in sharp contrast to classic cationic polymer delivery systems, where serum typically impairs the transfection efficiency [126]. The zwitterionic nature of the LAF-XP carriers at physiological pH suggests that they exhibit a different protein corona compared to purely polycationic delivery systems. In the case of LPEI, plasma proteins such as IgM, fibrinogen, fibronectin, and complement C3 were found to bind to non-PEGylated polyplexes [32]; furthermore it has been demonstrated that PEG shielding significantly reduces protein adsorption and improves in vivo performance of DNA polyplexes. To assess the effect of PEGylation on the protein corona composition of mRNA LAF-XP polyplexes, unmodified and PEGylated 1611 and 1752 (10% DSPE-PEG-N3) were incubated in serum, and unbound proteins were removed via centrifugation. Finally, SDS-PAGE with subsequent Coomassie staining was performed (imperial protein stain) (Figure S10a).

The 1611 and 1752 polyplexes exhibited distinct differences in protein adsorption. Unmodified 1611 showed higher protein adsorption compared to unmodified 1752. For both carriers, PEGylation with 10% DSPE-PEG-N3 visibly reduced protein adsorption. To quantify these differences, the intensities of the three most prominent protein bands (~70 kDa, 15 kDa, and 8 kDa) were analyzed using ImageJ. Quantitative analysis revealed that unmodified 1752 adsorbed less protein than 1611 across all bands. This difference likely derives from their distinct chemical composition. Carrier 1752, rich in LAF motifs, is less cationic, whereas 1611 has fewer LAF moieties and more resembles classical cationic delivery systems such as LPEI. The reduced protein adsorption of the 1752 LAF-XP mRNA polyplexes suggests that the LAF motif exerts a shielding effect. PEGylation further reduced protein adsorption in both carriers (Figure S10b).

Subsequently, the protein coronas of 1611 and 1752 mRNA polyplexes without or with PEGylation (3% DMG-PEG) were investigated in more detail using proteomic methods. This methodological approach allowed the detection of differences in the composition of the protein corona, whereas quantitative changes in the total amount of protein cannot be resolved.

**Figure 5:**
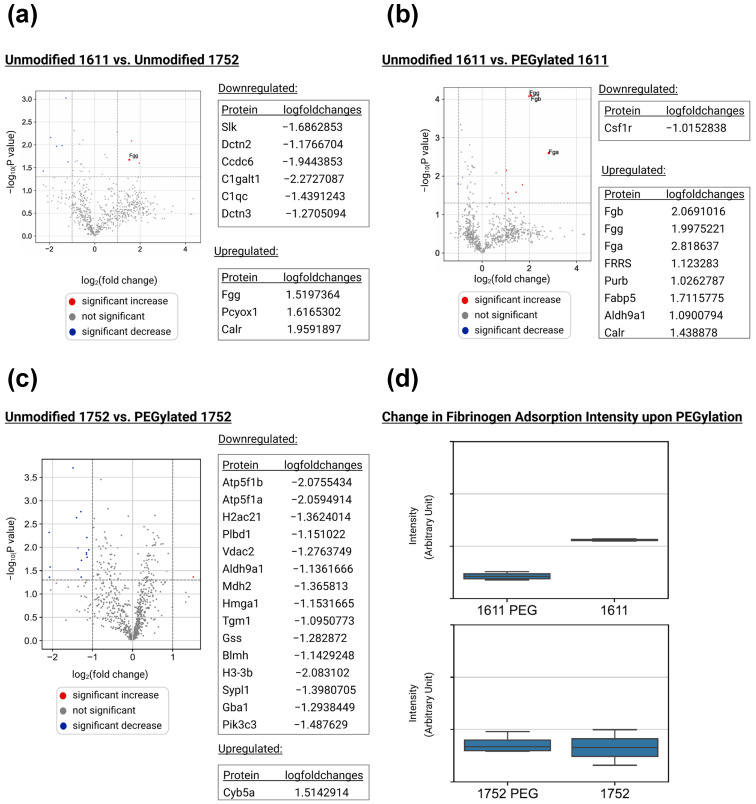
Proteomic analysis on the protein corona of LAF-XP mRNA polyplexes. PEGylated 1611 (1611 PEG) and 1752 (1752 PEG) refer to formulations containing 3% DMG-PEG. Volcano plots of (**a**) unmodified 1611 compared to unmodified 1752, displaying proteins up- and downregulated for 1611 relative to 1752; (**b**) unmodified 1611 compared to PEGylated 1611, displaying proteins up- and downregulated for 1611 relative to PEGylated 1611; and (**c**) unmodified 1752 compared to PEGylated 1752, displaying proteins up- and downregulated for 1752 relative to PEGylated 1752. (**d**) Intensity of fibrinogen adsorption of unmodified and PEGylated 1611 and 1752 mRNA LAF-XP polyplexes.

Initial analysis of the unmodified carrier polyplexes suggests that fibrinogen (Fgg) was upregulated in 1611 compared to 1752 (Figure 5a). Fibrinogen is a large, negatively charged glycoprotein. It is known to bind strongly to positively charged particles. Carrier 1611 exhibits a zeta potential of 25–35 mV, whereas 1752 shows a markedly lower zeta potential of approximately 10 (Figure 1a). Upon PEGylation with 3% DMG-PEG, the zeta potential of 1611 is decreased to ~13. PEGylated 1611 polyplexes thus show a zeta potential comparable to unmodified 1752 polyplexes (Figure 1a). As a result, PEGylated 1611 nanoparticles exhibit significantly reduced fibrinogen adsorption (Figure 5b,d). In contrast, 1752 polyplexes inherently adsorb less fibrinogen. Here PEGylation did not further decrease fibrinogen adsorption (Figure 5c,d). This difference is likely attributed to the distinct structural composition of the carriers. Carrier 1752 contains an Stp/LAF ratio of 1:4, whereas 1611 has a ratio of 1:2. The higher Stp content in 1611 likely contributes to its higher zeta potential. The cationic polar Stp unit is a structural derivative of LPEI, which is also known to strongly adsorb fibrinogen. PEGylation of highly cationic LPEI polyplexes likewise resulted in less fibrinogen adsorption [32]. In contrast, the amphiphilic LAF domain behaves differently. It is largely neutral and hydrophobic at physiological pH. In 1752, it likely accounts for its reduced zeta potential by partially neutralizing its surface charge. The decrease in fibrinogen adsorption implies that the LAF motif may provide a shielding effect.

### 3.6. Steric Stabilization of LAF-XP mRNA Polyplexes Against Protein- and Serum-Induced Aggregation Through PEGylation

Extensive research especially on cationic carrier systems has shown aggregation after intravenous application, resulting in toxicity [127]. Moreover, organ-specific targeting can be hampered. For instance, LPEI/DNA polyplexes at high doses aggregate with blood components, which passively accumulate in the lung, predominantly leading to high transfection of pulmonary tissue. This also poses a serious threat to biosafety. In the case of PEI, conjugation with PEG evaded these problems [24,32,128].

Initially, the stability of unmodified polyplexes with carriers 1611, 1752, and 1621 was investigated in the presence of negatively charged human transferrin (Figure S11a). Unmodified LAF-XP polyplexes were formulated according to the standard preparation protocol, followed by the addition of varying molar amounts of human transferrin (molar equivalent refers to the molar ratio of human transferrin to the carrier). Interestingly, contrary to the PBS results, 1611 appeared to be less stable than the bundles. It failed to form stable particles at any molar ratio of human transferrin. Carriers 1621 and 1752 formed stable particles at a molar equivalent of 0.25. As all carriers underwent aggregation at a molar ratio of 0.1 eq hTF, this ratio was chosen to investigate the impact of PEGylation.

The results were consistent with all carriers. PEGylation effectively prevented aggregation. Particles were slightly larger at 1.5% DMG-PEG compared to 3% DMG-PEG (Figure S11b). Minimal PEGylation again showed a great improvement in colloidal stability.

After intravenous application, besides ionic stress and protein adsorption, higher body temperature and shear forces may challenge the stability of the carriers even further [113,129,130,131]. Therefore, a serum assay was conducted. Here, LAF-XP polyplexes were tested in the presence of electrolytes, proteins, higher temperature, and shear forces all at once. Unmodified and PEGylated (3% and 10% DSPE-PEG-N3/DMG-PEG) LAF-XP mRNA polyplexes, formulated at in vivo concentration (10 µg/150 µL), were incubated in 90% fetal bovine serum for 2 h under continuous shaking conditions at 300 rpm at 37 °C. Subsequently, serum-incubated LAF-XP polyplexes were tested regarding their size and transfection efficiency.

The first DLS measurements were conducted. Prior to measurement, serum-incubated polyplexes were further diluted with HBG, resulting in a final serum concentration of 45%. Subsequently DLS measurements were performed. Unmodified carriers showed aggregation after 2 h (Figure S12b). Especially, the bundles 1621 and 1752 benefited from PEGylation. Both PEG lipids, DMG-PEG and DSPE-PEG-N3, at molar ratios of 3% and 10%, effectively prevented aggregation. Interestingly, as in the stability study with human transferrin, 1611 polyplexes appeared to be less stable. In that case, DMG-PEG at any molar ratio failed to improve stability. Only DSPE-PEG-N3 prevented aggregation (Figure S12b).

**Figure 6:**
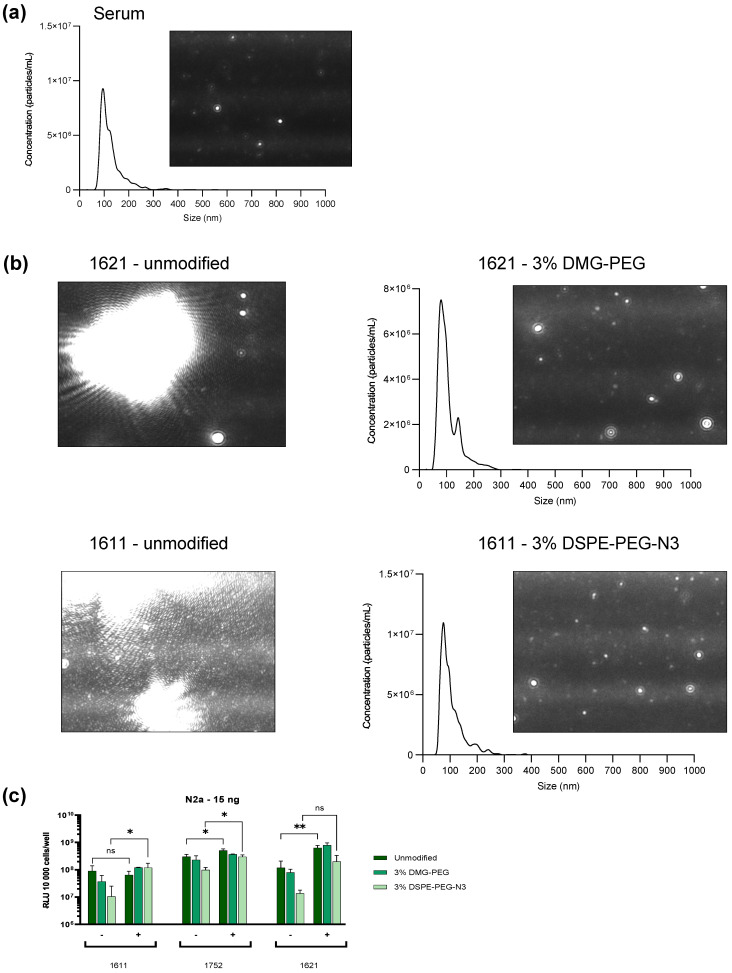
Assessment of stability and functionality in the presence of serum. Unmodified and PEGylated LAF-XP mRNA polyplexes were diluted and incubated in 90% fetal bovine serum (FBS) (1:10) for 2 h at 37 °C under continuous shaking conditions at 300 rpm. (**a**,**b**) NTA measurements with the corresponding NTA video frame of serum, unmodified, and PEGylated 1621 and 1611 LAF-XP mRNA polyplexes (3% DMG-PEG/DSPE-PEG-N3). For NTA measurements, FBS-incubated samples were further diluted 1:200 in Hepes 7.4 (**c**) Transfection efficiency of unmodified and PEGylated LAF-XP polyplexes (3% DMG-PEG/DSPE) following dilution and incubation in 90% serum was compared to the corresponding LAF-XP polyplexes diluted in HBG (-) in N2a cells. Luciferase gene expression was determined after 24 h at a dose of 15 ng mRNA-LUC per well (10,000 cells/well) (*n* = 3; mean ± SD). The statistical significance was determined by an unpaired *t*-test with Welch’s correction; ns, not significant; * *p* ≤ 0.05, ** *p* ≤ 0.01.

DLS measurements have limitations in polydisperse samples. Large aggregates might mask smaller particles, leading to underrepresentation of the smaller particle populations. Therefore, to further support these findings, nanoparticle tracking analysis (NTA) was performed. In order to distinguish the serum background from LAF-XP polyplexes, a serum blank was measured prior to the polyplex measurements (Figure 6a). The observed trends were very similar to the DLS results. The unmodified carriers exhibited aggregation after 2 h. They formed large, bright particles that prevented precise NTA quantification (Figure 6b). For 1621, PEGylation greatly improved colloidal stability, with 3% DMG-PEG effectively preventing aggregation. Both PEG lipids, DMG-PEG and DSPE-PEG, enhanced particle stability (Figure 6b and Figure S13a). The 1611 polyplexes again appeared to be less stable. For DMG-PEG, particle stabilization was achieved only at a molar ratio of 10%, whereas 3% was insufficient (Figure S13b). In contrast, for DSPE-PEG-N3, 3% improved stability and prevented aggregation (Figure 6b).

These results indicate that bundle carriers 1621 and 1752 benefit from PEGylation to a greater extent than the U-shape 1611. This is likely due to their higher number in apolar LAF domains (1621 and 1752 B2 bundles—4 LAF domains, 1611 U-shape—2 LAF domains), in which the PEG lipids may be better anchored. This would also explain the superior stabilizing effect of DSPE-PEG-N3 compared to DMG-PEG in 1611 LAF-XP polyplexes.

Furthermore, transfection efficiency upon serum incubation was assessed. As a control, LAF-XP polyplexes were diluted in HBG instead of serum prior to transfection (Figure 6c). The DSL measurement has shown that 3% PEG resulted in stabilization. Therefore, only LAF-XP polyplexes, PEGylated at a molar ratio of 3% PEG-DSPE/DMG, were examined.

The luciferase expression assay revealed that the unmodified carriers were not negatively affected by serum incubation. In fact, for the bundles 1621 and 1752, serum incubation even enhanced transfection efficiency compared to their HBG diluted counterparts. On the other hand, the transfection efficiency of 1611 remained largely unchanged.

For bundle carriers 1621 and 1752, PEGylation under serum-free conditions resulted in a decrease in transfection efficiency with DSPE-PEG-N3 again having a stronger effect. In the presence of serum, however, this reduction in transfection efficiency was less pronounced. In fact, for 1621, 3% DMG-PEG enhanced the transfection efficiency.

Similarly, for 1611 under serum-free conditions, PEGylation led to a decrease in transfection efficiency. However, in the presence of serum, the trends were the opposite. PEGylation led to an increase in transfection efficiency. DSPE-PEG-N3 (containing a larger lipid anchor) outperformed DMG-PEG. This reversed trend might be explained by the prior DLS measurement, which revealed that DSPE-PEG-N3 at 3% managed to better stabilize the particles, which ultimately leads to enhanced transfection efficiency. Besides the different Stp/LAF ratio, the topology may also have a large impact on PEG anchoring. In the bundles, the LAF domains are forced to stay in close proximity as they are connected via lysine residues. In contrast, in 1611 the two LAF moieties are separated by the Stp unit. Bundles may more easily form more localized lipophilic regions in which PEG lipids are more stably anchored. Moreover, 1611 contains a primary amine in its backbone, which might interfere with lipophilic interactions between the LAF unit and the lipid anchors.

### 3.7. In Vivo Dose Study: 1621 and 1752

Carriers 1621 and 1752 greatly benefit from PEGylation and were therefore selected for intravenous application. A dose escalation study was conducted to evaluate whether minimal PEGylation (3% DMG-PEG) would allow the injection of a higher mRNA dose. Specifically, for 1621, a starting dose of 5 µg mRNA was selected (A/J mice), and for 1752, the starting dose was 6 µg mRNA (N2a tumor-bearing NMRI mice).

Upon injection of the high dose of unmodified 1621 and 1752 LAF-XP mRNA polyplexes, mice suffered from severe toxicity symptoms and had to be euthanized. The dose had to be reduced for the remaining animals and was then well tolerated. In contrast, the higher doses were tolerated with the PEGylated formulations, which exhibited no signs of toxicity.

These observations indicate that PEGylation may contribute to biosafety as its ability to prevent aggregation, thus improving stability, made it possible to increase the dose. Regarding transfection efficiency, there were no notable differences between the unmodified and the PEGylated formulations (Figure S14a,b).

To explore any effect of PEGylation on tumor accumulation, polyplexes of unmodified 1621 (at the low dose of 2.5 µg mRNA) and PEGylated 1621 (at the low and high doses of 2.5 µg and 5 µg mRNA) were intravenously injected into N2a tumor bearing A/J mice. The higher dose of 5 µg mRNA of the PEGylated formulation was again well tolerated, confirming the results from the initial dosing study (Figure S15). A comparison between the PEGylated and the unmodified formulation did not show notable differences in tumor expression signal or in other organs. These findings suggest that minimal PEGylation without targeting does not influence transfection efficiency but enhances biocompatibility.

## 4. Conclusions

Several studies have shown that modification of cationic nanoparticles with hydrophilic polymers such as PEG improves colloidal stability, reduces nonspecific interactions with plasma proteins, and also decreases toxicity in vivo. However, such shielding may inactivate nanosystems as PEG may also impair binding to the target cell surface and endosomal membrane, decreasing the transfection efficiency. As a compromise, often only a suboptimum percentage (around 2 molar %) of PEG shielding agent has been applied. Several strategies have been developed to address this so-called PEG dilemma [83,84,132]. Targeting ligands have been successfully introduced to reestablish target cell surface interaction and endocytosis. This, however, does not necessarily restore the lipid membrane interaction required of endosomal destabilization and release into the cytosol [83]. Endosomal cleavage of PEG from nanoparticles via acid-labile [133,134,135,136], reducible [137], or enzymatically cleavable [138] linkers was found to restore endosomal escape and efficacy. In this study we present an alternative reversible shielding strategy to evade the drawbacks of conventional PEGylation. DSPE-PEG-N3 and DMG-PEG are anchored within the LAF-XP polyplex via hydrophobic interactions between the lipid tail of PEG lipids and the hydrophobic LAF domain of the carrier. However, in contrast to standard LNPs or lipoplexes, upon endosomal protonation of the LAF tertiary amine, the lipidic character of the LAF domain switches to a polar state [71]. As a result, hydrophobic anchoring of the PEG lipids is reduced, and the PEG lipids are assumed to dissociate from the polyplex surface under endosomal conditions.

Compared with LNPs, PEGylated LAF-XP polyplexes can be formulated in a simplified manufacturing process, with PEG lipids co-diluted with the LAF-XP carrier at neutral pH in water and subsequently flash-mixing with nucleic acids. No additional dilution or dialysis steps are required. PEGylation had a clear impact on physicochemical properties, decreasing the zeta potential. At high molar ratios (10% and 20%) of DSPE-PEG-N3, effective surface shielding was achieved. The EGFR-binding peptide GE11 was conjugated via copper-free click chemistry between its DBCO group and the azido group on DSPE-PEG-N3. GE11-functionalized LAF-XP polyplexes efficiently restored transfection efficiency in EGFR-positive cell lines. This platform demonstrates high flexibility and can be extended to other ligands or targeting moieties. Furthermore, a reduction in zeta potential was particularly beneficial for the more positively charged carrier 1611, resulting in decreased fibrinogen binding. LAF-rich 1752, exhibiting a low zeta potential, inherently showed low fibrinogen binding. PEGylation did not further reduce adsorption.

Low molar ratios of DMG-PEG (1.5% and 3%) were suitable for improving colloidal stability. Highly effective lipophilic LAF-XP carriers, previously stable only in LNP formulations, were able to form well-defined particles upon inclusion of 1.5% DMG-PEG. Furthermore, 3% DMG-PEG effectively prevented aggregation in the presence of salts, proteins, and full serum, improving biosafety of LAF-XP mRNA polyplexes. These findings represent an important step toward targeted nucleic acid therapies.

## Data Availability

Data are contained within this article and the Supplementary Materials.

## Supplementary Materials

The following supporting information can be downloaded at: https://www.mdpi.com/article/10.3390/polym17222979/s1: Table S1: Overview of GE11-DBCO ligands and the corresponding mass analyses by MALDI-TOF-MS; Figure S1: Structure, MALDI-TOF-MS spectra and HPLC analysis of DBCO-GE11. [M+H]+ calculated 1854.84 [M+H]+ found 1851.03; Figure S2: Structure, MALDI-TOF-MS spectra and HPLC analysis of DBCO-scrGE11. [M+H]+ calculated 1854.84 [M+H]+ found 1851.27; Figure S3: Agarose gel shift. Variation in PEGylation (molar %) of LAF-XP mRNA polyplexes with DMG-PEG and DSPE-PEG-N3. Comparison of unmodified with PEGylated polyplexes; Figure S4: Variation in molar % PEGylation of LAF-XP mRNA polyplexes with DMG-PEG and DSPE-PEG-N3. (a) Gene transfer activity of unmodified PEGylated polyplexes in KB cells 24 h after transfection. Comparison of unmodified with PEGylated polyplexes at a dose of 15 ng mRNA-LUC/well. Transfection efficacy was determined by luciferase gene expression assay (n = 3, mean + SD). (b,c) Metabolic activity in relation to HBG-treated control cells determined by MTT assay on KB and N2a cells at a dose of 15 ng mRNA-LUC/well (n = 3, mean + SD). The statistical significance was determined by unpaired *t*-test with Welch’s correction; ns, not significant; **p* ≤ 0.05, ****p* ≤ 0.001, *****p* ≤ 0.0001; Figure S5: Unmodified (white) and PEGylated (blue) LAF-XP mRNA polyplexes were formulated and subsequently either diluted with HEPES buffer at pH 7.4 (non-dashed) or at pH 5.4 (dashed). After dilution, size and zeta potential were measured by DLS; Figure S6: Variation in molar % PEGylation of LAF-XP pDNA polyplexes with DMG-PEG and DSPE-PEG-N3. (a) Hydrodynamic sizes and polydispersity index (PDI) values of unmodified and PEGylated (different molar %) polyplexes, measured by dynamic light scattering (DLS) and (b) zeta potential analysis determined using electrophoretic light scattering (ELS). (c) Gene transfer activity of LPEI (200 ng pCMVLuc/well), unmodified and PEGylated polyplexes in N2a cells at 24 h after transfection. Comparison of unmodified with PEGylated polyplexes at a dose of 50 ng pCMVLuc/well. Transfection efficacy was determined by luciferase gene expression assay (n = 3, mean + SD). (d) Metabolic activity in relation to HBG treated control cells determined by MTT assay (n = 3, mean + SD). (e) Impact of PEGylation on compaction was determined via an ethidium bromide (EtBr) assay. EtBr fluorescence is proportional to the amount of free, non-compacted pDNA, as only unbound pDNA is accessible for EtBr intercalation. The statistical significance was determined by unpaired *t*-test with Welch’s correction; ns, not significant; **p* ≤ 0.05, ***p* ≤ 0.01; Figure S7: In vitro evaluation of GE11 functionalized 1611 LAF-XP pDNA polyplexes. (a) Z Average, polydispersity index (PDI) and Zeta potential of GE11 functionalized 1719 polyplexes determined by DLS and ELS. (b) Comparison of luciferase gene expression of LPEI (200 ng pCMVLuc/well), unmodified 1719 and PEGylated 1719 (25% DSPE-PEG-N3) and GE11 targeted 1719 (25% DSPE-PEG-GE11) polyplexes on HepG2 and HUH7 cells at a dose of 50 ng pCMVLuc/well after a total incubation time of 24 h (n = 3; mean + SD). The statistical significance was determined by unpaired *t*-test with Welch’s correction; ns, not significant; ***p* ≤ 0.01, ****p* ≤ 0.001; Figure S8: Kinetic study of the hydrodynamic size of unmodified and PEGylated (0.5% and 1% DMG-PEG) LAF-XP mRNA polyplexes in the presence of PBS. Prior to PBS addition, LAF-XP mRNA polyplexes were preincubated for 30 min under either acidic (pH 4) or physiological (pH 7.4) conditions at 37°C and shaking (300 rpm); Figure S9: Stabilization of B2 bundles 1621 and 1752 LAF-XP mRNA polyplexes at low NP ratio. Z average, polydispersity index (PDI) and zeta potential of unmodified and PEGylated (1.5% and 3% DMG-PEG) 1621 and 1752 polyplexes at an N/P ratio of 12, determined by dynamic light scattering (DLS) and electrophoretic light scattering (ELS); Figure S10: (a) SDS-PAGE with subsequent coomassie blue staining (imperial protein stain) of mouse serum incubated unmodified and PEGylated (10% DSPE-PEG-N3) LAF-XP mRNA polyplexes. (b) Band intensities were quantified using ImageJ. Quantification for the comparison of unmodified 1611 to unmodified 1752 is displayed as percentage change (% change) using the formula $\%\;change=100\times \left(1-\frac{Intensity\;1752}{Intensity\;1611}\right)$ and for the comparison of PEGylated polyplexes against unmodified polyplexes the formula $\%\;change=100\times \left(1-\frac{Intensity\;PEGylated}{Intensity\;unmodified}\right)$ was used. Three protein bands were quantified at approximately 70, 15 and 8 kDa; Figure S11: Stabilization of LAF-XP polyplexes against human transferrin (hTF) induced aggregation with DMG-PEG. (a) Z-average and PDI of 1611, 1621, and 1752 in the presence of varying molar equivalents (carrier/hTF molar ratio) of human transferrin, measured by DLS. (b) Z-average, polydispersity index (PDI), and zeta potential of unmodified and PEGylated (1.5% and 3% DMG-PEG) carriers, both in the presence and absence of 0.1 eq hTF; Figure S12: Assessment of particle stability in the presence of serum. Unmodified and PEGylated LAF-XP mRNA polyplexes were diluted and incubated in 90% fetal bovine serum (FBS) for 2 h at 37°C under continuous shaking at 300 rpm. (a) and (b) Z average of serum, unmodified and PEGylated LAF-XP polyplexes (3% and 10% DMG-PEG/DSPE) was determined using DLS. For DLS measurements, 40 µL of the FBS-incubated samples were further diluted with 40 µL of HBG, resulting in a final volume of 80 µL, and transferred to a folded capillary cell; Figure S13: Assessment of stability and functionality in the presence of serum. Unmodified and PEGylated LAF-XP mRNA polyplexes were diluted and incubated in 90% fetal bovine serum (FBS) for 2 h at 37°C under continuous shaking at 300 rpm. FBS-incubated samples were further diluted 1:200 in Hepes (7.4). (a) and (b) NTA measurements with the corresponding NTA video frame of unmodified and PEGylated 1621 and 1611 LAF-XP mRNA polyplexes (3% and 10% DMG-PEG/DSPE-PEG-N3); Figure S14: Dose study of 1621 and 1752 LAF-XP mRNA polyplexes. Ex vivo luciferase (LUC) assay of organs from A/J mice (1621) (a) and N2a tumor-bearing NMRI mice (1752) (b), 24 h post-administration (n = 3; mean + SD). Comparison of unmodified 1621 and 1752 polyplexes, both at NP 24, administered at doses of 2.5 µg (1621) and 3 µg (1752), to PEGylated 1621 (NP 24; 3% DMG-PEG) or 1752 (NP 24, 3% DMG-PEG) polyplexes at doses of 5 µg or 6 µg mRNA. Unmodified 1621 NP 24 and 1752 NP 24 at doses of 5 µg (1621) or 6 µg mRNA (1752) caused severe toxicity, resulting in indicated euthanasia shortly after administration; Figure S15: In vivo transfection efficiency assessment of unmodified 1621 vs. PEGylated 1621 LAF-XP mRNA polyplexes in N2a tumor-bearing A/J mice. Ex vivo luciferase (LUC) assay of organs comparing unmodified 1621 polyplexes at a dose of 2.5 µg mRNA with PEGylated (3% DMG-PEG) 1621 polyplexes at doses of 2.5 µg and 5 µg mRNA, 24 h post-administration (n = 3; mean ± SD).

## Abbreviations

The following abbreviations are used in this manuscript:

mRNA	Messenger ribonucleic acid
pDNA	Plasmid deoxyribonucleic acid
siRNA	Small interfering ribonucleic acid
DMG-PEG	1,2-Dimyristoyl-rac-glycero-3-methoxypolyethylene glycol-2k
DSPE-PEG-N3	1,2-Distearoyl-sn-glycero-3-phosphoethanolamine-N [azido (polyethylene glycol)-2k]
(SPAAC)	Strain-promoted azide-alkyne cycloaddition
DBCO	Dibenzocyclooctyne
LNP	Lipid nanoparticle
FDA	Food and Drug Administration
COVID-19	Coronavirus disease 2019
pHPMA	Poly(N-(2-hydroxypropyl)methacrylamide)
pOx	Poly(2-oxazoline)
apoE	Apolipoprotein E
LAF	Lipo-amino fatty acid
LAF-XP	Lipo-amino fatty acid–xenopeptide
SPPS	Solid-phase-assisted peptide synthesis
Stp	Succinoyl tetraethylene pentamine
EGFR	Epidermal growth factor receptor
HEPES	4-(2-Hydroxyethyl)-1-piperazineethansulfonic acid
HBG	HEPES-buffered glucose
PBS	Phosphate-buffered saline
DLS	Dynamic light scattering
N2A	Neuro-2a cell line
HEPG2	Human hepatocellular carcinoma cell line
HUH7	Human hepatoma cell line
PDI	Polydispersity index
MTT	3-(4,5-Dimethylthiazol-2-yl)-2,5-diphenyltetrazolium bromide
HPLC	High-performance liquid chromatography
MALDI-TOF-MS	Matrix-assisted laser desorption/ionization–time of flight–mass spectrometry
EtBr	Ethidium bromide
FGG	Fibrinogen
Csf1r	Colony stimulating factor 1 receptor
PEI	Polyethylenimine
NTA	Nanoparticle tracking analysis

## References

[1] Bulcha J.T., Wang Y., Ma H., Tai P.W.L., Gao G. (2021). Viral vector platforms within the gene therapy landscape. Signal Transduct. Target. Ther..

[2] Wang J.H., Gessler D.J., Zhan W., Gallagher T.L., Gao G. (2024). Adeno-associated virus as a delivery vector for gene therapy of human diseases. Signal Transduct. Target. Ther..

[3] Felgner P.L., Barenholz Y., Behr J.P., Cheng S.H., Cullis P., Huang L., Jessee J.A., Seymour L., Szoka F., Thierry A.R. (1997). Nomenclature for synthetic gene delivery systems. Hum. Gene Ther..

[4] van der Meel R., Wender P.A., Merkel O.M., Lostalé-Seijo I., Montenegro J., Miserez A., Laurent Q., Sleiman H., Luciani P. (2025). Next-generation materials for nucleic acid delivery. Nat. Rev. Mater..

[5] Witten J., Hu Y., Langer R., Anderson D.G. (2024). Recent advances in nanoparticulate RNA delivery systems. Proc. Natl. Acad. Sci. USA.

[6] Berger S., Lachelt U., Wagner E. (2024). Dynamic carriers for therapeutic RNA delivery. Proc. Natl. Acad. Sci. USA.

[7] Freitag F., Wagner E. (2021). Optimizing synthetic nucleic acid and protein nanocarriers: The chemical evolution approach. Adv. Drug Deliv. Rev..

[8] Luo T., Liang H., Jin R., Nie Y. (2019). Virus inspired and mimetic designs in nonviral gene delivery. J. Gene Med..

[9] Nabel G.J., Nabel E.G., Yang Z.Y., Fox B.A., Plautz G.E., Gao X., Huang L., Shu S., Gordon D., Chang A.E. (1993). Direct gene transfer with DNA-liposome complexes in melanoma: Expression, biologic activity, and lack of toxicity in humans. Proc. Natl. Acad. Sci. USA.

[10] Sakurai Y., Hatakeyama H., Sato Y., Hyodo M., Akita H., Harashima H. (2013). Gene silencing via RNAi and siRNA quantification in tumor tissue using mend, a liposomal siRNA delivery system. Mol. Ther..

[11] Anderson D.G., Lynn D.M., Langer R. (2003). Semi-automated synthesis and screening of a large library of degradable cationic polymers for gene delivery. Angew. Chem. Int. Ed. Engl..

[12] Itaka K., Harada A., Yamasaki Y., Nakamura K., Kawaguchi H., Kataoka K. (2004). In situ single cell observation by fluorescence resonance energy transfer reveals fast intra-cytoplasmic delivery and easy release of plasmid DNA complexed with linear polyethylenimine. J. Gene Med..

[13] Pack D.W., Hoffman A.S., Pun S., Stayton P.S. (2005). Design and development of polymers for gene delivery. Nat. Rev. Drug Discov..

[14] Miyata K., Nishiyama N., Kataoka K. (2012). Rational design of smart supramolecular assemblies for gene delivery: Chemical challenges in the creation of artificial viruses. Chem. Soc. Rev..

[15] Lachelt U., Wagner E. (2015). Nucleic acid therapeutics using polyplexes: A journey of 50 years (and beyond). Chem. Rev..

[16] Kumar R., Chalarca C.F.S., Bockman M.R., Bruggen C.V., Grimme C.J., Dalal R.J., Hanson M.G., Hexum J.K., Reineke T.M. (2021). Polymeric delivery of therapeutic nucleic acids. Chem. Rev..

[17] Dirisala A., Uchida S., Li J., Van Guyse J.F.R., Hayashi K., Vummaleti S.V.C., Kaur S., Mochida Y., Fukushima S., Kataoka K. (2022). Effective mrna protection by poly(l-ornithine) synergizes with endosomal escape functionality of a charge-conversion polymer toward maximizing mrna introduction efficiency. Macromol. Rapid Commun..

[18] Cullis P.R., Felgner P.L. (2024). The 60-year evolution of lipid nanoparticles for nucleic acid delivery. Nat. Rev. Drug Discov..

[19] Lin Y., Li M., Luo Z., Meng Y., Zong Y., Ren H., Yu X., Tan X., Liu F., Wei T. (2025). Tissue-specific mRNA delivery and prime editing with peptide-ionizable lipid nanoparticles. Nat. Mater..

[20] Adams D., Gonzalez-Duarte A., O’Riordan W.D., Yang C.C., Ueda M., Kristen A.V., Tournev I., Schmidt H.H., Coelho T., Berk J.L. (2018). Patisiran, an RNAi therapeutic, for hereditary transthyretin amyloidosis. N. Engl. J. Med..

[21] Verbeke R., Lentacker I., De Smedt S.C., Dewitte H. (2021). The dawn of mRNA vaccines: The COVID-19 case. J. Control. Release.

[22] Schoenmaker L., Witzigmann D., Kulkarni J.A., Verbeke R., Kersten G., Jiskoot W., Crommelin D.J.A. (2021). mRNA-lipid nanoparticle COVID-19 vaccines: Structure and stability. Int. J. Pharm..

[23] Gillmore J.D., Gane E., Taubel J., Pilebro B., Echaniz-Laguna A., Kao J., Litchy W., Shahda S., Haagensen A., Walsh L. (2025). Nexiguran ziclumeran gene editing in hereditary ATTR with polyneuropathy. N. Engl. J. Med..

[24] Chollet P., Favrot M.C., Hurbin A., Coll J.L. (2002). Side-effects of a systemic injection of linear polyethylenimine-DNA complexes. J. Gene Med..

[25] Plank C., Mechtler K., Szoka F.C., Wagner E. (1996). Activation of the complement system by synthetic DNA complexes: A potential barrier for intravenous gene delivery. Hum. Gene Ther..

[26] Merkel O.M., Urbanics R., Bedocs P., Rozsnyay Z., Rosivall L., Toth M., Kissel T., Szebeni J. (2011). In vitro and in vivo complement activation and related anaphylactic effects associated with polyethylenimine and polyethylenimine-graft-poly(ethylene glycol) block copolymers. Biomaterials.

[27] Mosqueira V.C.F., Legrand P., Gulik A., Bourdon O., Gref R., Labarre D., Barratt G. (2001). Relationship between complement activation, cellular uptake and surface physicochemical aspects of novel peg-modified nanocapsules. Biomaterials.

[28] Wen P., Ke W., Dirisala A., Toh K., Tanaka M., Li J. (2023). Stealth and pseudo-stealth nanocarriers. Adv. Drug Deliv. Rev..

[29] Hong K., Zheng W., Baker A., Papahadjopoulos D. (1997). Stabilization of cationic liposome-plasmid DNA complexes by polyamines and poly(ethylene glycol)-phospholipid conjugates for efficient in vivo gene delivery. FEBS Lett..

[30] Wheeler J.J., Palmer L., Ossanlou M., MacLachlan I., Graham R.W., Zhang Y.P., Hope M.J., Scherrer P., Cullis P.R. (1999). Stabilized plasmid-lipid particles: Construction and characterization. Gene Ther..

[31] Katayose S., Kataoka K. (1997). Water-soluble polyion complex associates of DNA and poly(ethylene glycol)-poly(l-lysine) block copolymer. Bioconjug Chem..

[32] Ogris M., Brunner S., Schuller S., Kircheis R., Wagner E. (1999). Pegylated DNA/transferrin-PEI complexes: Reduced interaction with blood components, extended circulation in blood and potential for systemic gene delivery. Gene Ther..

[33] Itaka K., Yamauchi K., Harada A., Nakamura K., Kawaguchi H., Kataoka K. (2003). Polyion complex micelles from plasmid DNA and poly(ethylene glycol)-poly(l-lysine) block copolymer as serum-tolerable polyplex system: Physicochemical properties of micelles relevant to gene transfection efficiency. Biomaterials.

[34] Tockary T.A., Osada K., Chen Q., Machitani K., Dirisala A., Uchida S., Nomoto T., Toh K., Matsumoto Y., Itaka K. (2013). Tethered peg crowdedness determining shape and blood circulation profile of polyplex micelle gene carriers. Macromolecules.

[35] Roy P., Kreofsky N.W., Chalarca C.F.S., Reineke T.M. (2025). Binary copolymer blending enhances pDNA delivery performance and colloidal shelf stability of quinine-based polyplexes. Bioconjug. Chem..

[36] Katayose S., Kataoka K. (1998). Remarkable increase in nuclease resistance of plasmid DNA through supramolecular assembly with poly(ethylene glycol)-poly(l-lysine) block copolymer. J. Pharm. Sci..

[37] Erbacher P., Bettinger T., Belguise-Valladier P., Zou S., Coll J.L., Behr J.P., Remy J.S. (1999). Transfection and physical properties of various saccharide, poly(ethylene glycol), and antibody-derivatized polyethylenimines (PEI). J. Gene Med..

[38] Merdan T., Kunath K., Petersen H., Bakowsky U., Voigt K.H., Kopecek J., Kissel T. (2005). Pegylation of poly(ethylene imine) affects stability of complexes with plasmid DNA under in vivo conditions in a dose-dependent manner after intravenous injection into mice. Bioconjug. Chem..

[39] Dash P.R., Read M.L., Fisher K.D., Howard K.A., Wolfert M., Oupicky D., Subr V., Strohalm J., Ulbrich K., Seymour L.W. (2000). Decreased binding to proteins and cells of polymeric gene delivery vectors surface modified with a multivalent hydrophilic polymer and retargeting through attachment of transferrin. J. Biol. Chem..

[40] Burke R.S., Pun S.H. (2010). Synthesis and characterization of biodegradable hpma-oligolysine copolymers for improved gene delivery. Bioconjug. Chem..

[41] Gaspar V.M., Goncalves C., de Melo-Diogo D., Costa E.C., Queiroz J.A., Pichon C., Sousa F., Correia I.J. (2014). Poly(2-ethyl-2-oxazoline)-PLA-g-PEI amphiphilic triblock micelles for co-delivery of minicircle DNA and chemotherapeutics. J. Control. Release.

[42] Yamaleyeva D.N., Makita N., Hwang D., Haney M.J., Jordan R., Kabanov A.V. (2023). Poly(2-oxazoline)-based polyplexes as a peg-free plasmid DNA delivery platform. Macromol. Biosci..

[43] Heller P., Birke A., Huesmann D., Weber B., Fischer K., Reske-Kunz A., Bros M., Barz M. (2014). Introducing peptoplexes: Polylysine-block-polysarcosine based polyplexes for transfection of HEK 293T cells. Macromol. Biosci..

[44] Klein P.M., Klinker K., Zhang W., Kern S., Kessel E., Wagner E., Barz M. (2018). Efficient shielding of polyplexes using heterotelechelic polysarcosines. Polymers.

[45] Bayraktutan H., Kopiasz R.J., Elsherbeny A., Espuga M.M., Gumus N., Oz U.C., Polra K., McKay P.F., Shattock R.J., Ordóñez-Morán P. (2024). Polysarcosine functionalised cationic polyesters efficiently deliver self-amplifying mRNA. Polym. Chem..

[46] Hou X., Zaks T., Langer R., Dong Y. (2021). Lipid nanoparticles for mRNA delivery. Nat. Rev. Mater..

[47] Kim J., Eygeris Y., Gupta M., Sahay G. (2021). Self-assembled mRNA vaccines. Adv. Drug Deliv. Rev..

[48] Lokugamage M.P., Vanover D., Beyersdorf J., Hatit M.Z.C., Rotolo L., Echeverri E.S., Peck H.E., Ni H., Yoon J.K., Kim Y. (2021). Optimization of lipid nanoparticles for the delivery of nebulized therapeutic mRNA to the lungs. Nat. Biomed. Eng..

[49] Hald Albertsen C., Kulkarni J.A., Witzigmann D., Lind M., Petersson K., Simonsen J.B. (2022). The role of lipid components in lipid nanoparticles for vaccines and gene therapy. Adv. Drug Deliv. Rev..

[50] Klibanov A.L., Maruyama K., Torchilin V.P., Huang L. (1990). Amphipathic polyethyleneglycols effectively prolong the circulation time of liposomes. FEBS Lett..

[51] Allen T.M., Hansen C., Martin F., Redemann C., Yau-Young A. (1991). Liposomes containing synthetic lipid derivatives of poly(ethylene glycol) show prolonged circulation half-lives in vivo. Biochim. Biophys. Acta.

[52] Lasic D.D., Martin F.J., Gabizon A., Huang S.K., Papahadjopoulos D. (1991). Sterically stabilized liposomes: A hypothesis on the molecular origin of the extended circulation times. Biochim. Biophys. Acta.

[53] Papahadjopoulos D., Allen T.M., Gabizon A., Mayhew E., Matthay K., Huang S.K., Lee K.D., Woodle M.C., Lasic D.D., Redemann C. (1991). Sterically stabilized liposomes: Improvements in pharmacokinetics and antitumor therapeutic efficacy. Proc. Natl. Acad. Sci. USA.

[54] Monck M.A., Mori A., Lee D., Tam P., Wheeler J.J., Cullis P.R., Scherrer P. (2000). Stabilized plasmid-lipid particles: Pharmacokinetics and plasmid delivery to distal tumors following intravenous injection. J. Drug Target..

[55] Tranchant I., Thompson B., Nicolazzi C., Mignet N., Scherman D. (2004). Physicochemical optimisation of plasmid delivery by cationic lipids. J. Gene Med..

[56] Gabizon A., Catane R., Uziely B., Kaufman B., Safra T., Cohen R., Martin F., Huang A., Barenholz Y. (1994). Prolonged circulation time and enhanced accumulation in malignant exudates of doxorubicin encapsulated in polyethylene-glycol coated liposomes. Cancer Res..

[57] Meyer O., Kirpotin D., Hong K., Sternberg B., Park J.W., Woodle M.C., Papahadjopoulos D. (1998). Cationic liposomes coated with polyethylene glycol as carriers for oligonucleotides. J. Biol. Chem..

[58] Akinc A., Querbes W., De S., Qin J., Frank-Kamenetsky M., Jayaprakash K.N., Jayaraman M., Rajeev K.G., Cantley W.L., Dorkin J.R. (2010). Targeted delivery of RNAi therapeutics with endogenous and exogenous ligand-based mechanisms. Mol. Ther..

[59] Chen S., Tam Y.Y.C., Lin P.J.C., Sung M.M.H., Tam Y.K., Cullis P.R. (2016). Influence of particle size on the in vivo potency of lipid nanoparticle formulations of siRNA. J. Control. Release.

[60] Harvie P., Wong F.M., Bally M.B. (2000). Use of poly(ethylene glycol)-lipid conjugates to regulate the surface attributes and transfection activity of lipid-DNA particles. J. Pharm. Sci..

[61] Mui B.L., Tam Y.K., Jayaraman M., Ansell S.M., Du X., Tam Y.Y., Lin P.J., Chen S., Narayanannair J.K., Rajeev K.G. (2013). Influence of polyethylene glycol lipid desorption rates on pharmacokinetics and pharmacodynamics of siRNA lipid nanoparticles. Mol. Ther. Nucleic Acids.

[62] Waggoner L.E., Miyasaki K.F., Kwon E.J. (2023). Analysis of PEG-lipid anchor length on lipid nanoparticle pharmacokinetics and activity in a mouse model of traumatic brain injury. Biomater. Sci..

[63] Suzuki T., Suzuki Y., Hihara T., Kubara K., Kondo K., Hyodo K., Yamazaki K., Ishida T., Ishihara H. (2020). PEG shedding-rate-dependent blood clearance of PEGylated lipid nanoparticles in mice: Faster PEG shedding attenuates anti-PEG IgM production. Int. J. Pharm..

[64] Katakowski J.A., Mukherjee G., Wilner S.E., Maier K.E., Harrison M.T., DiLorenzo T.P., Levy M., Palliser D. (2016). Delivery of sirnas to dendritic cells using DEC205-targeted lipid nanoparticles to inhibit immune responses. Mol. Ther..

[65] Parhiz H., Shuvaev V.V., Pardi N., Khoshnejad M., Kiseleva R.Y., Brenner J.S., Uhler T., Tuyishime S., Mui B.L., Tam Y.K. (2018). Pecam-1 directed re-targeting of exogenous mRNA providing two orders of magnitude enhancement of vascular delivery and expression in lungs independent of apolipoprotein e-mediated uptake. J. Control. Release.

[66] Veiga N., Goldsmith M., Diesendruck Y., Ramishetti S., Rosenblum D., Elinav E., Behlke M.A., Benhar I., Peer D. (2019). Leukocyte-specific siRNA delivery revealing IRF8 as a potential anti-inflammatory target. J. Control. Release.

[67] Rosenblum D., Gutkin A., Kedmi R., Ramishetti S., Veiga N., Jacobi A.M., Schubert M.S., Friedmann-Morvinski D., Cohen Z.R., Behlke M.A. (2020). CRISPR-Cas9 genome editing using targeted lipid nanoparticles for cancer therapy. Sci. Adv..

[68] Tombácz I., Laczkó D., Shahnawaz H., Muramatsu H., Natesan A., Yadegari A., Papp T.E., Alameh M.G., Shuvaev V., Mui B.L. (2021). Highly efficient CD4+ T cell targeting and genetic recombination using engineered CD4+ cell-homing mRNA-LNPs. Mol. Ther..

[69] Escudé Martinez de Castilla P., Verdi V., de Voogt W., Sentí M.E., Koekman A.C., Rietveld J., van Kempen S., Yang Q., van Merris J., Jenster G. (2025). Nanobody-decorated lipid nanoparticles for enhanced mRNA delivery to tumors in vivo. Adv. Healthc. Mater..

[70] Papp T.E., Zeng J., Shahnawaz H., Akyianu A., Breda L., Yadegari A., Steward J., Shi R., Li Q., Mui B.L. (2025). CD47 peptide-cloaked lipid nanoparticles promote cell-specific mRNA delivery. Mol. Ther..

[71] Thalmayr S., Grau M., Peng L., Pöhmerer J., Wilk U., Folda P., Yazdi M., Weidinger E., Burghardt T., Höhn M. (2023). Molecular chameleon carriers for nucleic acid delivery: The sweet spot between lipoplexes and polyplexes. Adv. Mater..

[72] Germer J., Lessl A.L., Pöhmerer J., Grau M., Weidinger E., Höhn M., Yazdi M., Cappelluti M.A., Lombardo A., Lächelt U. (2024). Lipo-xenopeptide polyplexes for CRISPR/Cas9 based gene editing at ultra-low dose. J. Control. Release.

[73] Yazdi M., Pohmerer J., Kafshgari M.H., Seidl J., Grau M., Hohn M., Vetter V., Hoch C.C., Wollenberg B., Multhoff G. (2024). In vivo endothelial cell gene silencing by siRNA-LNPs tuned with lipoamino bundle chemical and ligand targeting. Small.

[74] Haase F., Pöhmerer J., Yazdi M., Grau M., Zeyn Y., Wilk U., Burghardt T., Höhn M., Hieber C., Bros M. (2024). Lipoamino bundle LNPs for efficient mRNA transfection of dendritic cells and macrophages show high spleen selectivity. Eur. J. Pharm. Biopharm..

[75] Philipp J., Dabkowska A., Reiser A., Frank K., Krzyszton R., Brummer C., Nickel B., Blanchet C.E., Sudarsan A., Ibrahim M. (2023). pH-dependent structural transitions in cationic ionizable lipid mesophases are critical for lipid nanoparticle function. Proc. Natl. Acad. Sci. USA.

[76] Yanez Arteta M., Kjellman T., Bartesaghi S., Wallin S., Wu X., Kvist A.J., Dabkowska A., Szekely N., Radulescu A., Bergenholtz J. (2018). Successful reprogramming of cellular protein production through mRNA delivered by functionalized lipid nanoparticles. Proc. Natl. Acad. Sci. USA.

[77] Rodl W., Schaffert D., Wagner E., Ogris M. (2013). Synthesis of polyethylenimine-based nanocarriers for systemic tumor targeting of nucleic acids. Methods Mol. Biol..

[78] Steffens R.C., Thalmayr S., Weidinger E., Seidl J., Folda P., Hohn M., Wagner E. (2024). Modulating efficacy and cytotoxicity of lipoamino fatty acid nucleic acid carriers using disulfide or hydrophobic spacers. Nanoscale.

[79] Morys S., Levacic A.K., Urnauer S., Kempter S., Kern S., Radler J.O., Spitzweg C., Lachelt U., Wagner E. (2017). Influence of defined hydrophilic blocks within oligoaminoamide copolymers: Compaction versus shielding of pdna nanoparticles. Polymers.

[80] Smith R.J., Beck R.W., Prevette L.E. (2015). Impact of molecular weight and degree of conjugation on the thermodynamics of DNA complexation and stability of polyethylenimine-graft-poly(ethylene glycol) copolymers. Biophys. Chem..

[81] Koning G.A., Morselt H.W., Kamps J.A., Scherphof G.L. (2001). Uptake and intracellular processing of peg-liposomes and peg-immunoliposomes by kupffer cells in vitro. J. Liposome Res..

[82] Mishra S., Webster P., Davis M.E. (2004). Pegylation significantly affects cellular uptake and intracellular trafficking of non-viral gene delivery particles. Eur.J Cell Biol..

[83] Walker G.F., Fella C., Pelisek J., Fahrmeir J., Boeckle S., Ogris M., Wagner E. (2005). Toward synthetic viruses: Endosomal pH-triggered deshielding of targeted polyplexes greatly enhances gene transfer in vitro and in vivo. Mol. Ther..

[84] Wang T., Upponi J.R., Torchilin V.P. (2012). Design of multifunctional non-viral gene vectors to overcome physiological barriers: Dilemmas and strategies. Int. J. Pharm..

[85] Lai T.C., Kataoka K., Kwon G.S. (2011). Pluronic-based cationic block copolymer for forming pDNA polyplexes with enhanced cellular uptake and improved transfection efficiency. Biomaterials.

[86] Vetter V.C., Wagner E. (2022). Targeting nucleic acid-based therapeutics to tumors: Challenges and strategies for polyplexes. J. Control. Release.

[87] Chen J., Gamou S., Takayanagi A., Shimizu N. (1994). A novel gene delivery system using EGF receptor-mediated endocytosis. FEBS Lett..

[88] Kircheis R., Ostermann E., Wolschek M.F., Lichtenberger C., Magin-Lachmann C., Wightman L., Kursa M., Wagner E. (2002). Tumor-targeted gene delivery of tumor necrosis factor-alpha induces tumor necrosis and tumor regression without systemic toxicity. Cancer. Gene Ther..

[89] Bellocq N.C., Pun S.H., Jensen G.S., Davis M.E. (2003). Transferrin-containing, cyclodextrin polymer-based particles for tumor-targeted gene delivery. Bioconjug. Chem..

[90] Merdan T., Callahan J., Petersen H., Kunath K., Bakowsky U., Kopeckova P., Kissel T., Kopecek J. (2003). Pegylated polyethylenimine-fab’ antibody fragment conjugates for targeted gene delivery to human ovarian carcinoma cells. Bioconjug. Chem..

[91] Oba M., Fukushima S., Kanayama N., Aoyagi K., Nishiyama N., Koyama H., Kataoka K. (2007). Cyclic RGD peptide-conjugated polyplex micelles as a targetable gene delivery system directed to cells possessing α_v_β_3_ and α_v_β_5_ integrins. Bioconjug. Chem..

[92] Oba M., Aoyagi K., Miyata K., Matsumoto Y., Itaka K., Nishiyama N., Yamasaki Y., Koyama H., Kataoka K. (2008). Polyplex micelles with cyclic RGD peptide ligands and disulfide cross-links directing to the enhanced transfection via controlled intracellular trafficking. Mol. Pharm..

[93] Liu L., Zheng M., Librizzi D., Renette T., Merkel O.M., Kissel T. (2016). Efficient and tumor targeted sirna delivery by polyethylenimine-graft-polycaprolactone-block-poly(ethylene glycol)-folate (pei-pcl-peg-fol). Mol. Pharm..

[94] Kim M.W., Jeong H.Y., Kang S.J., Choi M.J., You Y.M., Im C.S., Lee T.S., Song I.H., Lee C.G., Rhee K.J. (2017). Cancer-targeted nucleic acid delivery and quantum dot imaging using EGF receptor aptamer-conjugated lipid nanoparticles. Sci. Rep..

[95] Mickler F.M., Mockl L., Ruthardt N., Ogris M., Wagner E., Brauchle C. (2012). Tuning nanoparticle uptake: Live-cell imaging reveals two distinct endocytosis mechanisms mediated by natural and artificial EGFR targeting ligand. Nano Lett..

[96] Ren H., Zhou L., Liu M., Lu W., Gao C. (2015). Peptide GE11–polyethylene glycol–polyethylenimine for targeted gene delivery in laryngeal cancer. Med. Oncol..

[97] Lee D., Lee Y.M., Kim J., Lee M.K., Kim W.J. (2015). Enhanced tumor-targeted gene delivery by bioreducible polyethylenimine tethering EGFR divalent ligands. Biomater. Sci..

[98] Schmohl K.A., Gupta A., Grunwald G.K., Trajkovic-Arsic M., Klutz K., Braren R., Schwaiger M., Nelson P.J., Ogris M., Wagner E. (2017). Imaging and targeted therapy of pancreatic ductal adenocarcinoma using the theranostic sodium iodide symporter (NIS) gene. Oncotarget.

[99] Wang Y., Luo J., Truebenbach I., Reinhard S., Klein P.M., Höhn M., Kern S., Morys S., Loy D.M., Wagner E. (2020). Double click-functionalized siRNA polyplexes for gene silencing in epidermal growth factor receptor-positive tumor cells. ACS Biomater. Sci. Eng..

[100] Yu L., Wang Q., Wong R.C.H., Zhao S., Ng D.K.P., Lo P.-C. (2019). Synthesis and biological evaluation of phthalocyanine-peptide conjugate for EGFR-targeted photodynamic therapy and bioimaging. Dye. Pigment..

[101] Kursa M., Walker G.F., Roessler V., Ogris M., Roedl W., Kircheis R., Wagner E. (2003). Novel shielded transferrin-polyethylene glycol-polyethylenimine/DNA complexes for systemic tumor-targeted gene transfer. Bioconjug. Chem..

[102] Maurstad G., Stokke B.T., Varum K.M., Strand S.P. (2013). Pegylated chitosan complexes DNA while improving polyplex colloidal stability and gene transfection efficiency. Carbohydr. Polym..

[103] Tang M.X., Szoka F.C. (1997). The influence of polymer structure on the interactions of cationic polymers with DNA and morphology of the resulting complexes. Gene Ther..

[104] Needham D., McIntosh T.J., Lasic D.D. (1992). Repulsive interactions and mechanical stability of polymer-grafted lipid membranes. Biochim. Biophys. Acta.

[105] Oupicky D., Ogris M., Howard K.A., Dash P.R., Ulbrich K., Seymour L.W. (2002). Importance of lateral and steric stabilization of polyelectrolyte gene delivery vectors for extended systemic circulation. Mol. Ther..

[106] Pun S.H., Davis M.E. (2002). Development of a nonviral gene delivery vehicle for systemic application. Bioconjugate Chem..

[107] Vader P., van der Aa L.J., Engbersen J.F., Storm G., Schiffelers R.M. (2012). Physicochemical and biological evaluation of siRNA polyplexes based on pegylated poly(amido amine)s. Pharm. Res..

[108] Adolph E.J., Nelson C.E., Werfel T.A., Guo R., Davidson J.M., Guelcher S.A., Duvall C.L. (2014). Enhanced performance of plasmid DNA polyplexes stabilized by a combination of core hydrophobicity and surface pegylation. J. Mater. Chem. B.

[109] Wu T., Wang L., Ding S., You Y. (2017). Fluorinated PEG-polypeptide polyplex micelles have good serum-resistance and low cytotoxicity for gene delivery. Macromol. Biosci..

[110] Paunovska K., Sago C.D., Monaco C.M., Hudson W.H., Castro M.G., Rudoltz T.G., Kalathoor S., Vanover D.A., Santangelo P.J., Ahmed R. (2018). A direct comparison of in vitro and in vivo nucleic acid delivery mediated by hundreds of nanoparticles reveals a weak correlation. Nano Lett..

[111] Paunovska K., Loughrey D., Sago C.D., Langer R., Dahlman J.E. (2019). Using large datasets to understand nanotechnology. Adv. Mater..

[112] Huayamares S.G., Lokugamage M.P., Rab R., Da Silva Sanchez A.J., Kim H., Radmand A., Loughrey D., Lian L., Hou Y., Achyut B.R. (2023). High-throughput screens identify a lipid nanoparticle that preferentially delivers mRNA to human tumors in vivo. J. Control. Release.

[113] Berger S., Berger M., Bantz C., Maskos M., Wagner E. (2022). Performance of nanoparticles for biomedical applications: The in vitro/in vivo discrepancy. Biophys. Rev..

[114] Dilliard S.A., Sun Y., Brown M.O., Sung Y.C., Chatterjee S., Farbiak L., Vaidya A., Lian X., Wang X., Lemoff A. (2023). The interplay of quaternary ammonium lipid structure and protein corona on lung-specific mRNA delivery by selective organ targeting (sort) nanoparticles. J. Control. Release.

[115] Alberg I., Kramer S., Schinnerer M., Hu Q., Seidl C., Leps C., Drude N., Möckel D., Rijcken C., Lammers T. (2020). Polymeric nanoparticles with neglectable protein corona. Small.

[116] Zhang X., Si S., Lieberwirth I., Landfester K., Mailänder V. (2025). Engineered protein corona sustains stealth functionality of nanocarriers in plasma. J. Nano Biotechnol..

[117] Voke E., Arral M., Squire H.J., Lin T.J., Coreas R., Lui A., Iavarone A.T., Pinals R.L., Whitehead K.A., Landry M. (2025). Protein corona formed on lipid nanoparticles compromises delivery efficiency of mRNA cargo. Nat. Commun..

[118] Rademacker S., Carneiro S.P., Molbay M., Catapano F., Forné I., Imhof A., Wibel R., Heidecke C., Hölig P., Merkel O.M. (2025). The impact of lipid compositions on siRNA and mRNA lipid nanoparticle performance for pulmonary delivery. Eur. J. Pharm. Sci..

[119] He X., Wang R., Cao Y., Ding Y., Chang Y., Dong H., Xie R., Zhong G., Yang H., Li J. (2025). Lung-specific mrna delivery by ionizable lipids with defined structure-function relationship and unique protein corona feature. Adv. Sci..

[120] van Straten D., Sork H., van de Schepop L., Frunt R., Ezzat K., Schiffelers R.M. (2024). Biofluid specific protein coronas affect lipid nanoparticle behavior in vitro. J. Control. Release.

[121] Lundqvist M., Stigler J., Elia G., Lynch I., Cedervall T., Dawson K.A. (2008). Nanoparticle size and surface properties determine the protein corona with possible implications for biological impacts. Proc. Natl. Acad. Sci. USA.

[122] Lundqvist M., Stigler J., Cedervall T., Berggard T., Flanagan M.B., Lynch I., Elia G., Dawson K. (2011). The evolution of the protein corona around nanoparticles: A test study. ACS Nano.

[123] Cedervall T., Lynch I., Lindman S., Berggard T., Thulin E., Nilsson H., Dawson K.A., Linse S. (2007). Understanding the nanoparticle-protein corona using methods to quantify exchange rates and affinities of proteins for nanoparticles. Proc. Natl. Acad. Sci. USA.

[124] Vilanova O., Mittag J.J., Kelly P.M., Milani S., Dawson K.A., Radler J.O., Franzese G. (2016). Understanding the kinetics of protein-nanoparticle corona formation. ACS Nano.

[125] Milani S., Bombelli F.B., Pitek A.S., Dawson K.A., Radler J. (2012). Reversible versus irreversible binding of transferrin to polystyrene nanoparticles: Soft and hard corona. ACS Nano.

[126] Zelphati O., Uyechi L.S., Barron L.G., Szoka F.C. (1998). Effect of serum components on the physico-chemical properties of cationic lipid/oligonucleotide complexes and on their interactions with cells. Biochim. Biophys. Acta.

[127] Senior J.H., Trimble K.R., Maskiewicz R. (1991). Interaction of positively-charged liposomes with blood: Implications for their application in vivo. Biochim. Biophys. Acta.

[128] Casper J., Schenk S.H., Parhizkar E., Detampel P., Dehshahri A., Huwyler J. (2023). Polyethylenimine (PEI) in gene therapy: Current status and clinical applications. J. Control. Release.

[129] Takeda K.M., Yamasaki Y., Dirisala A., Ikeda S., Tockary T.A., Toh K., Osada K., Kataoka K. (2017). Effect of shear stress on structure and function of polyplex micelles from poly(ethylene glycol)-poly(l-lysine) block copolymers as systemic gene delivery carrier. Biomaterials.

[130] Yin D., Wen H., Wu G., Li S., Liu C., Lu H., Liang D. (2020). Pegylated gene carriers in serum under shear flow. Soft Matter.

[131] Wen H., Yu Q., Yin Y., Pan W., Yang S., Liang D. (2017). Shear effects on stability of DNA complexes in the presence of serum. Biomacromolecules.

[132] Hatakeyama H., Akita H., Harashima H. (2011). A multifunctional envelope type nano device (MEND) for gene delivery to tumours based on the EPR effect: A strategy for overcoming the PEG dilemma. Adv. Drug Deliv. Rev..

[133] Guo X., MacKay J.A., Szoka F.C. (2003). Mechanism of pH-triggered collapse of phosphatidylethanolamine liposomes stabilized by an ortho ester polyethyleneglycol lipid. Biophys. J..

[134] Murthy N., Campbell J., Fausto N., Hoffman A.S., Stayton P.S. (2003). Design and synthesis of pH-responsive polymeric carriers that target uptake and enhance the intracellular delivery of oligonucleotides. J. Control. Release.

[135] Fella C., Walker G.F., Ogris M., Wagner E. (2008). Amine-reactive pyridylhydrazone-based PEG reagents for pH-reversible PEI polyplex shielding. Eur. J. Pharm. Sci..

[136] Nie Y., Günther M., Gu Z., Wagner E. (2011). Pyridylhydrazone-based PEGylation for pH-reversible lipopolyplex shielding. Biomaterials.

[137] Takae S., Miyata K., Oba M., Ishii T., Nishiyama N., Itaka K., Yamasaki Y., Koyama H., Kataoka K. (2008). PEG-detachable polyplex micelles based on disulfide-linked block catiomers as bioresponsive nonviral gene vectors. J. Am. Chem. Soc..

[138] Hatakeyama H., Akita H., Kogure K., Oishi M., Nagasaki Y., Kihira Y., Ueno M., Kobayashi H., Kikuchi H., Harashima H. (2007). Development of a novel systemic gene delivery system for cancer therapy with a tumor-specific cleavable PEG-lipid. Gene Ther..

