# Supplementary material for: PEGylation Enhances Colloidal Stability and Promotes Ligand-Mediated Targeting of LAF–Xenopeptide mRNA Complexes
Source: Polymers (Basel). 2025 Nov 9;17(22):2979. doi: 10.3390/polym17222979 (PMC12656170; doi:10.3390/polym17222979)
Supplement: Supplementary file 1 [file polymers-17-02979-s001.zip › polymers-3931320-supplementary.pdf]

Supporting Information

PEGylation Enhances Colloidal Stability and Promotes Ligand-Mediated Targeting of LAF–Xenopeptide mRNA Complexes

Paul Folda [1,2], Eric Weidinger [1,2], Johanna Seidl [1], Mina Yazdi [1], Jana Pöhmerer [1], Melina Grau [1], David P. Minde [3], Mayar Ali [3], Ceren Kimna [3,*] and Ernst Wagner [1,2,*]

[1] Pharmaceutical Biotechnology, Department of Pharmacy, Ludwig-Maximilians-Universität (LMU) Munich, 81377 Munich, Germany; paul.folda@cup.uni-muenchen.de (P.F.); eric.weidinger@cup.uni-muenchen.de (E.We.); johanna.seidl@cup.uni-muenchen.de (J.S.); mina.yazdi@cup.uni-muenchen.de (M.Y.); jana.poehmerer@cup.uni-muenchen.de (J.P.); melina.grau@cup.uni-muenchen.de (M.G.)

[2] Center for NanoScience (CeNS), LMU Munich, 80799 Munich, Germany

[3] Institute for Intelligent Biotechnologies (iBIO), Helmholtz Center Munich, 85764 Neuherberg, Germany; david.minde@helmholtz-munich.de (D.P.M.); mayar.ali@helmholtz-munich.de (M.A.)

* Correspondence: ceren.kimna@helmholtz-munich.de (C.K.); ernst.wagner@cup.uni-muenchen.de (E.W.)

S1. Supporting Figures and Tables

	Sequence	Calculated molecular weight [DA]	Mass by MALDI-TOF
**GE11-DBCO**	H_2N-YHWYGYTPQNVI-COOH	1854.84	1851.03
**GE11scr-DBCO**	H_2N-YWGPNIHYYTQV-COOH	1854.84	1851.27

Table S1. Overview of GE11-DBCO ligands and the corresponding mass analyses by MALDI-TOF-MS

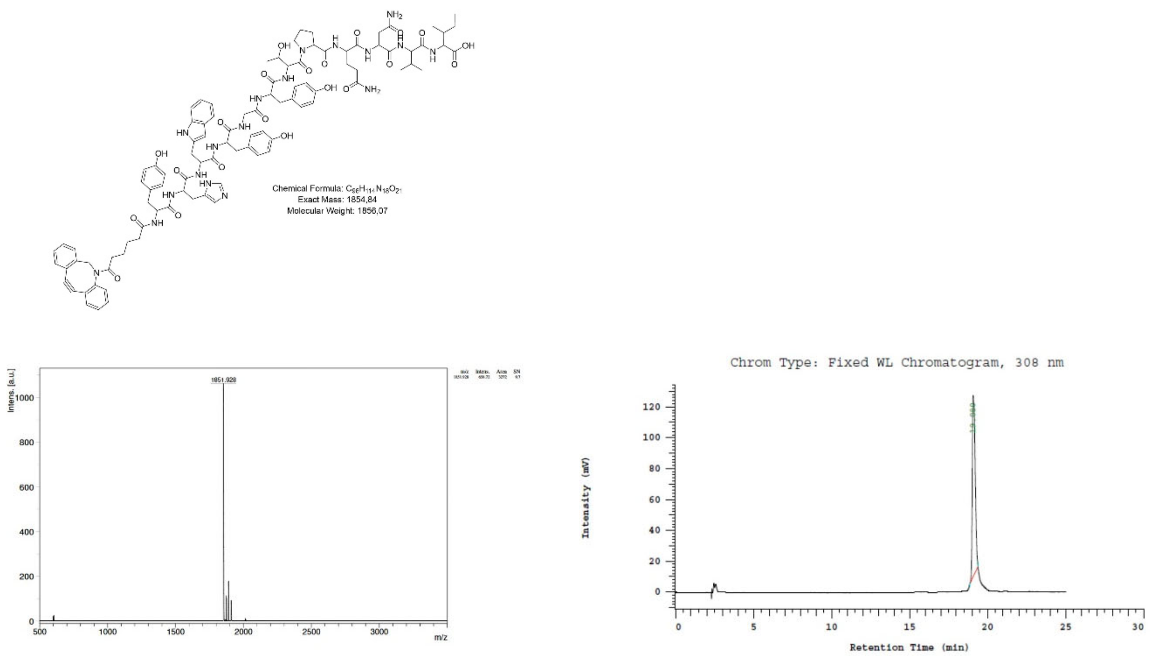

Figure S1. Structure, MALDI-TOF-MS spectra and HPLC analysis of DBCO-GE11**.** [M+H]+ calculated 1854.84 [M+H]+ found 1851.03

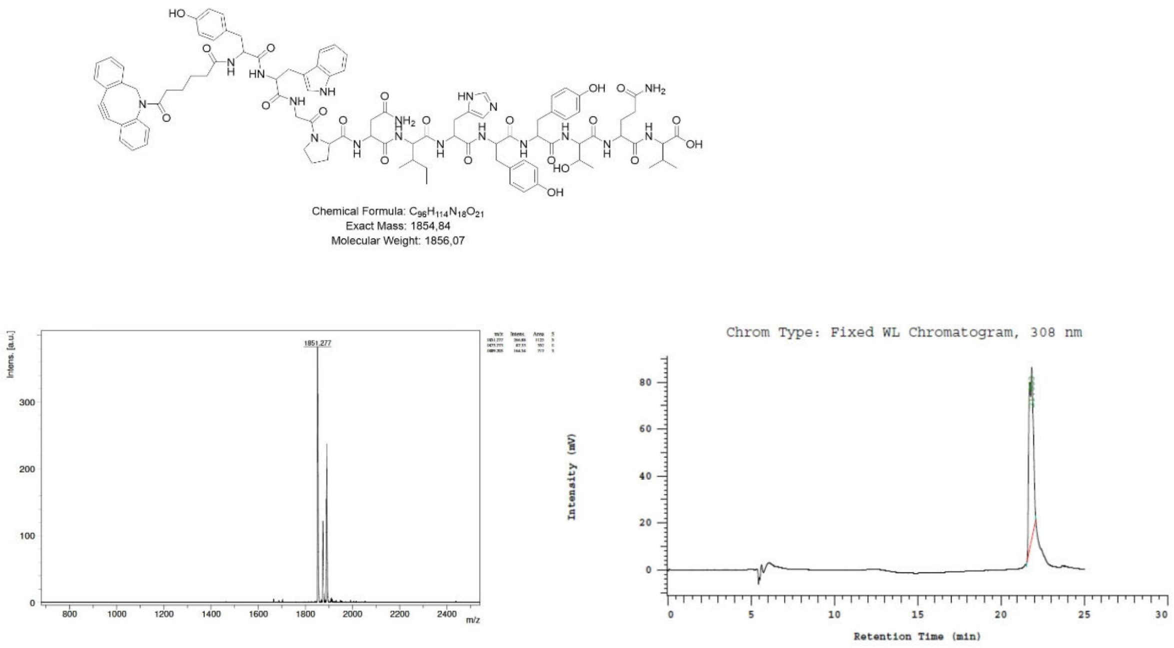

Figure S2. Structure, MALDI-TOF-MS spectra and HPLC analysis of DBCO-scrGE11. [M+H]+ calculated 1854.84 [M+H]+ found 1851.27

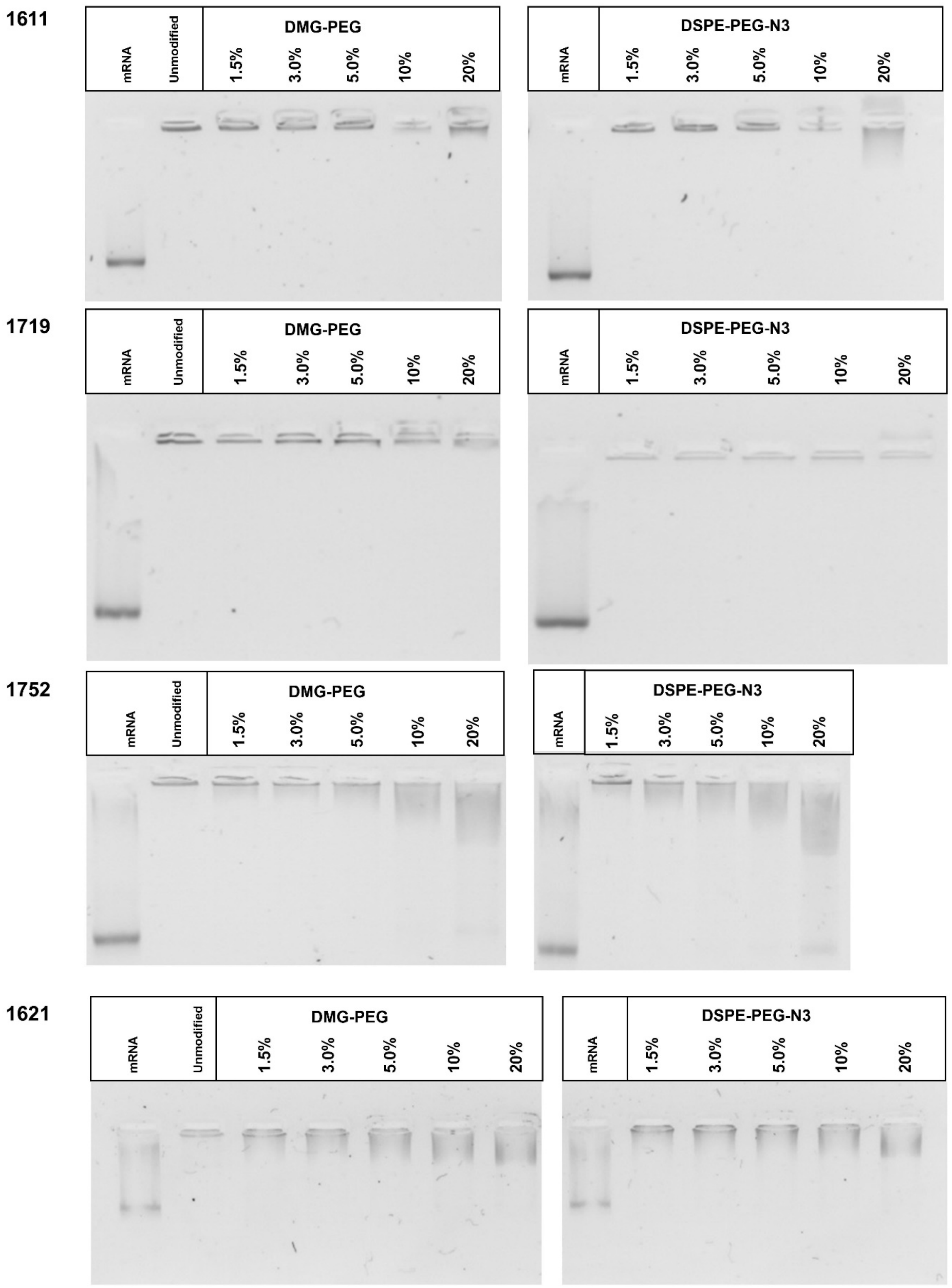

Figure S3. Agarose gel shift. Variation in PEGylation (molar %) of LAF-XP mRNA polyplexes with DMG-PEG and DSPE-PEG-N3. Comparison of unmodified with PEGylated polyplexes.

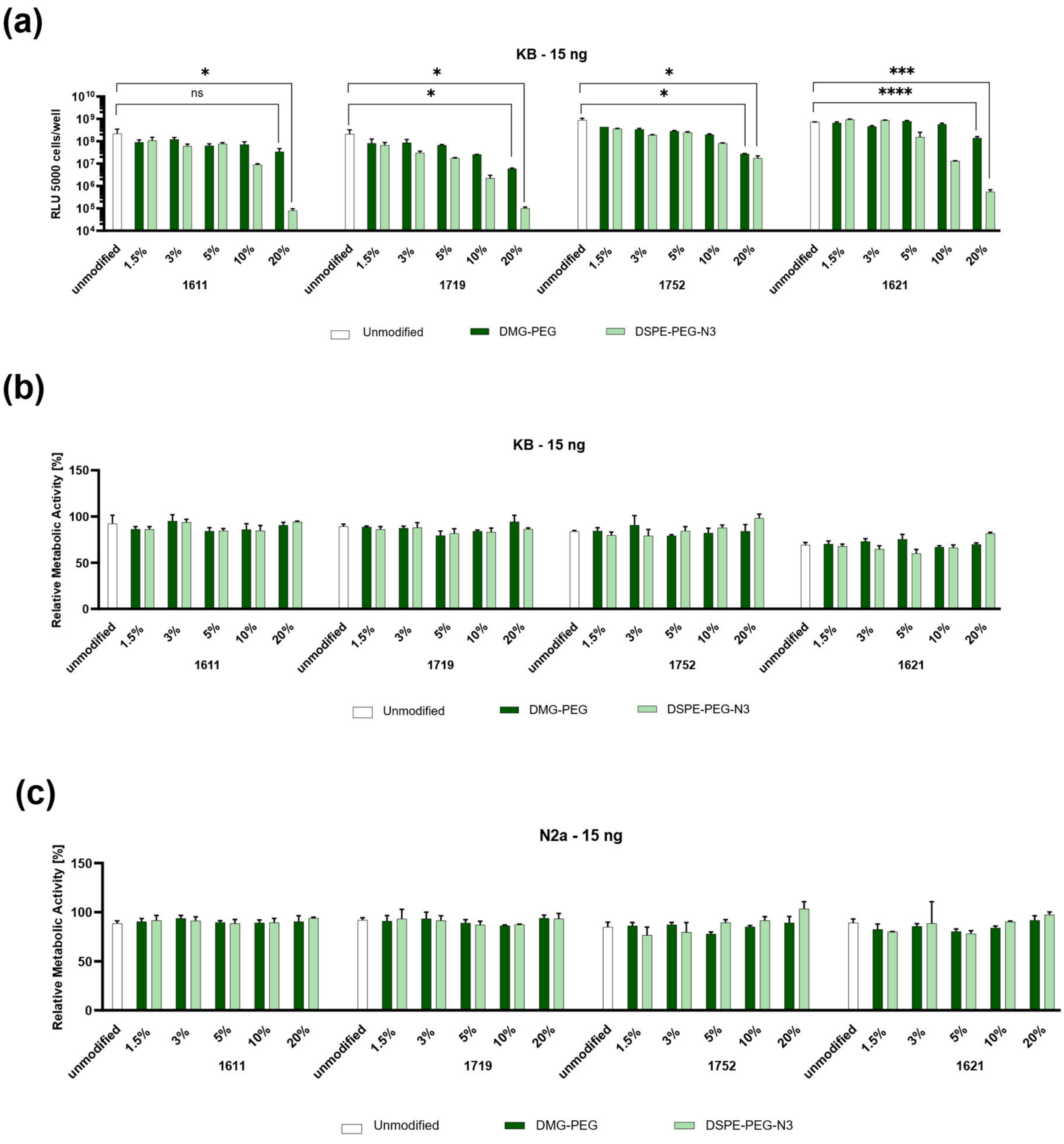

Figure S4. Variation in molar % PEGylation of LAF-XP mRNA polyplexes with DMG-PEG and DSPE-PEG-N3. **(a)** Gene transfer activity of unmodified PEGylated polyplexes in KB cells 24 h after transfection. Comparison of unmodified with PEGylated polyplexes at a dose of 15 ng mRNA-LUC/well. Transfection efficacy was determined by luciferase gene expression assay (n = 3, mean + SD). **(b) (c)** Metabolic activity in relation to HBG-treated control cells determined by MTT assay on KB and N2a cells at a dose of 15 ng mRNA-LUC/well (n = 3, mean + SD). The statistical significance was determined by unpaired t-test with Welch's correction; ns, not significant; *p ≤ 0.05, ***p ≤ 0.001, ****p ≤0.0001.

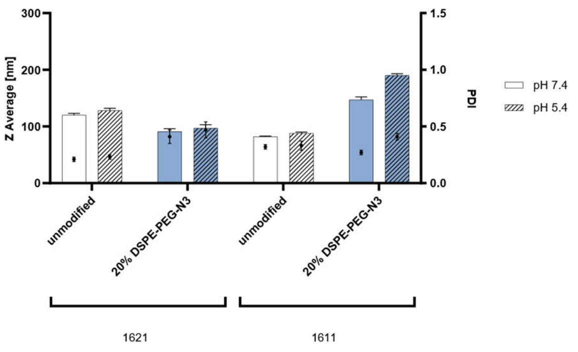

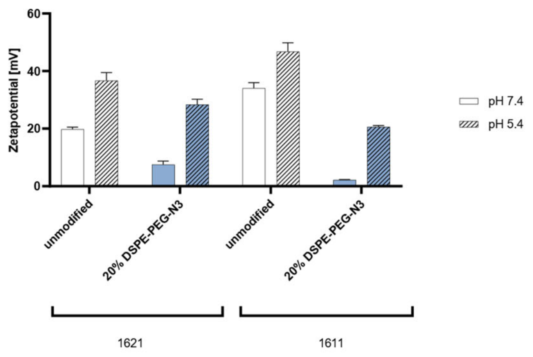

Figure S5. Unmodified (white) and PEGylated (blue) LAF-XP mRNA polyplexes were formulated and subsequently either diluted with HEPES buffer at pH 7.4 (non-dashed) or at pH 5.4 (dashed). After dilution, size and zeta potential were measured by DLS.

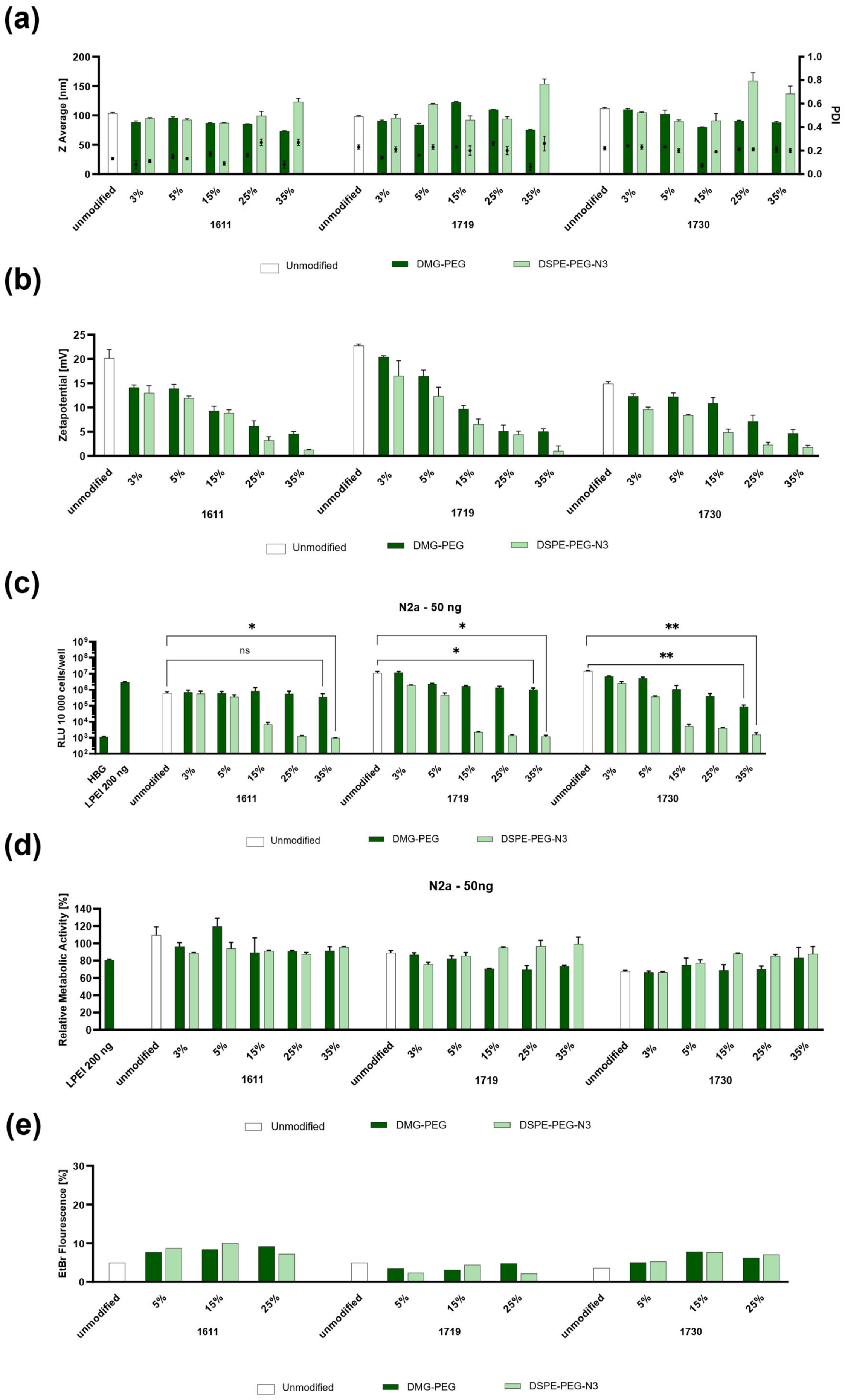
(a)
Z Average [nm]
PDI
unmodified
3%
5%
15%
25%
35%
1611
1719
1730
Unmodified
DMG-PEG
DSPE-PEG-N3
(b)
Zetapotential [mV]
(c)
N2a - 50 ng
RLU 10 000 cells/well
HBG
LPEI 200 ng
ns
*
**
(d)
N2a - 50ng
Relative Metabolic Activity [%]
(e)
EtBr Flourescence [%]

Figure S6. Variation in molar % PEGylation of LAF-XP pDNA polyplexes with DMG-PEG and DSPE-PEG-N3. **(a)** Hydrodynamic sizes and polydispersity index (PDI) values of unmodified and PEGylated (different molar %) polyplexes, measured by dynamic light scattering (DLS) and **(b)** zeta potential analysis determined using electrophoretic light scattering (ELS). **(c)** Gene transfer activity of LPEI (200 ng pCMVLuc/well), unmodified and PEGylated polyplexes in N2a cells at 24 h after transfection. Comparison of unmodified with PEGylated polyplexes at a dose of 50 ng pCMVLuc/well. Transfection efficacy was determined by luciferase gene expression assay (n = 3, mean + SD). **(d)** Metabolic activity in relation to HBG treated control cells determined by MTT assay (n = 3, mean + SD). **(e)** Impact of PEGylation on compaction was determined via an ethidium bromide (EtBr) assay. EtBr fluorescence is proportional to the amount of free, non-compacted pDNA, as only unbound pDNA is accessible for EtBr intercalation. The statistical significance was determined by unpaired t-test with Welch's correction; ns, not significant; *p ≤ 0.05, **p ≤ 0.01.

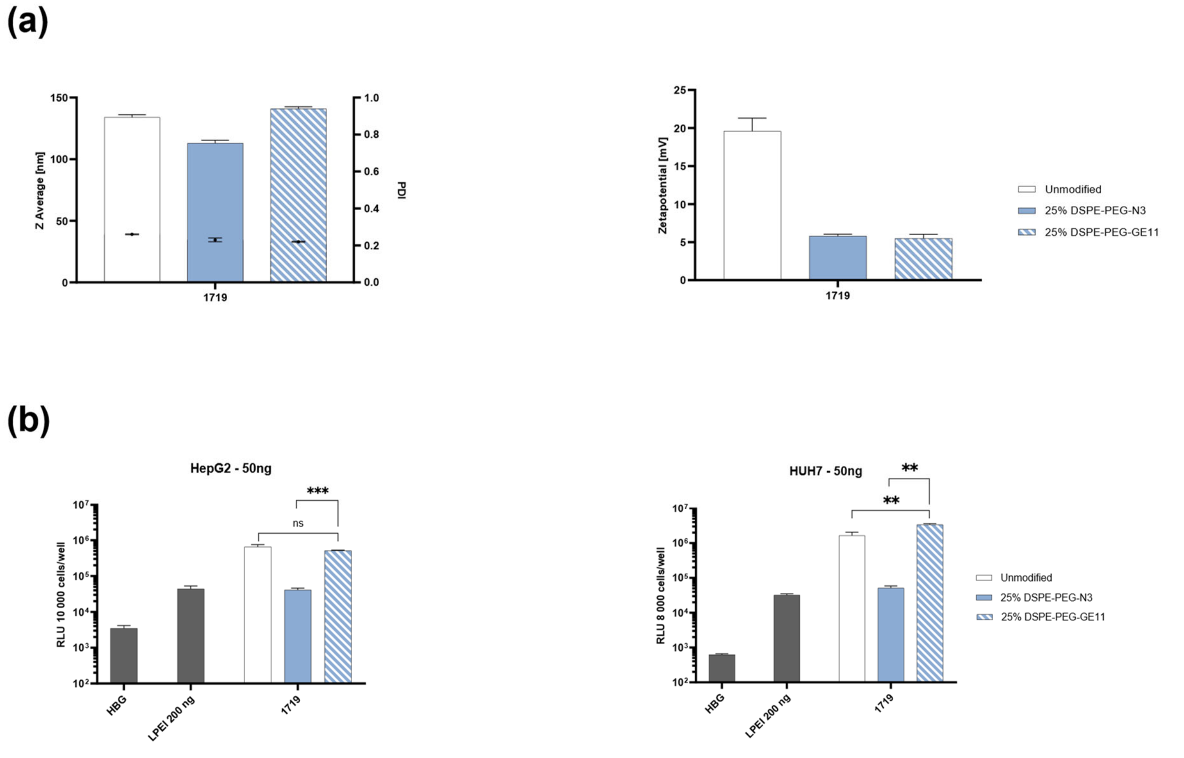

Figure S7. *In vitro* evaluation of GE11 functionalized 1611 LAF-XP pDNA polyplexes. **(a)** Z Average, polydispersity index (PDI) and Zeta potential of GE11 functionalized 1719 polyplexes determined by DLS and ELS. **(b)** Comparison of luciferase gene expression of LPEI (200 ng pCMVLuc/well), unmodified 1719 and PEGylated 1719 (25% DSPE-PEG-N3) and GE11 targeted 1719 (25% DSPE-PEG-GE11) polyplexes on HepG2 and HUH7 cells at a dose of 50 ng pCMVLuc/well after a total incubation time of 24 h (n=3; mean +SD). The statistical significance was determined by unpaired t-test with Welch's correction; ns, not significant; **p ≤ 0.01, ***p ≤ 0.001.

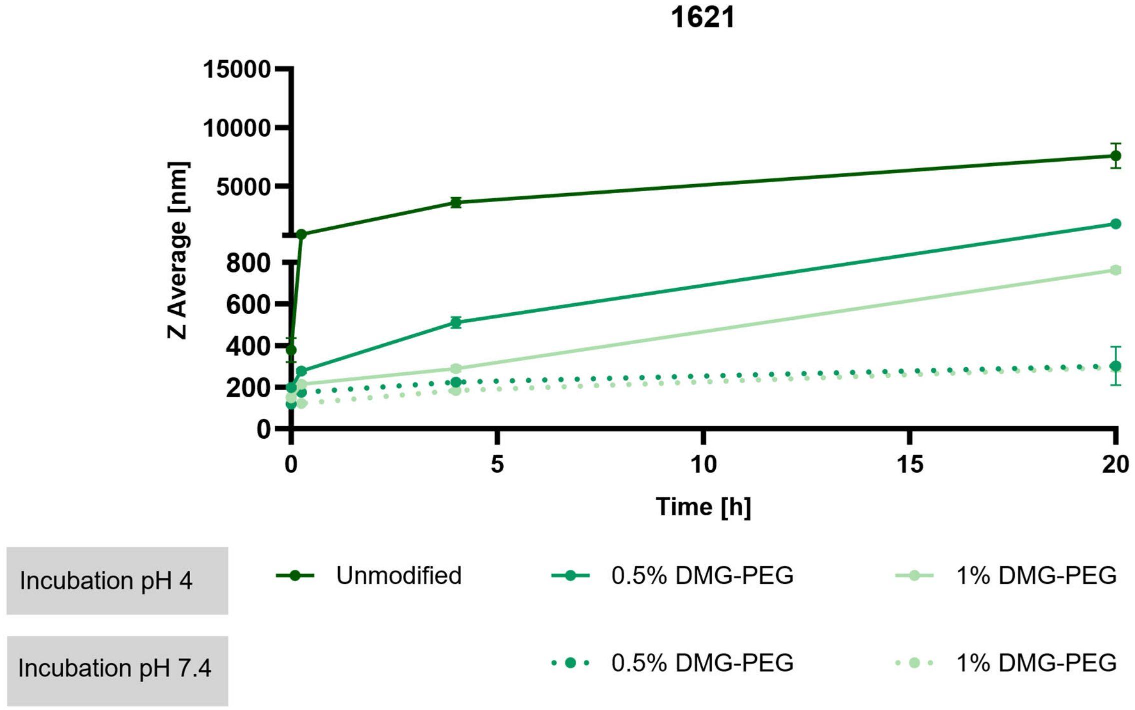

Figure S8. Kinetic study of the hydrodynamic size of unmodified and PEGylated (0.5% and 1% DMG-PEG) LAF-XP mRNA polyplexes in the presence of PBS. Prior to PBS addition, LAF-XP mRNA polyplexes were preincubated for 30 min under either acidic (pH 4) or physiological (pH 7.4) conditions at 37°C and shaking (300 rpm).

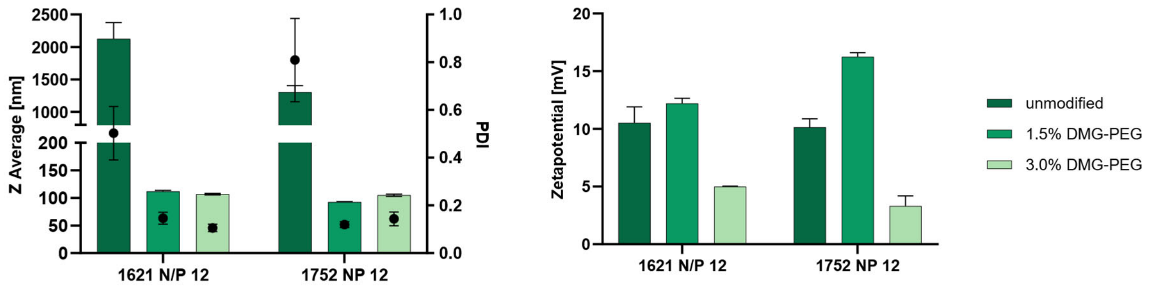

Figure S9. Stabilization of B2 bundles 1621 and 1752 LAF-XP mRNA polyplexes at low NP ratio. Z average, polydispersity index (PDI) and zeta potential of unmodified and PEGylated (1.5% and 3% DMG-PEG) 1621 and 1752 polyplexes at an N/P ratio of 12, determined by dynamic light scattering (DLS) and electrophoretic light scattering (ELS).

(a)

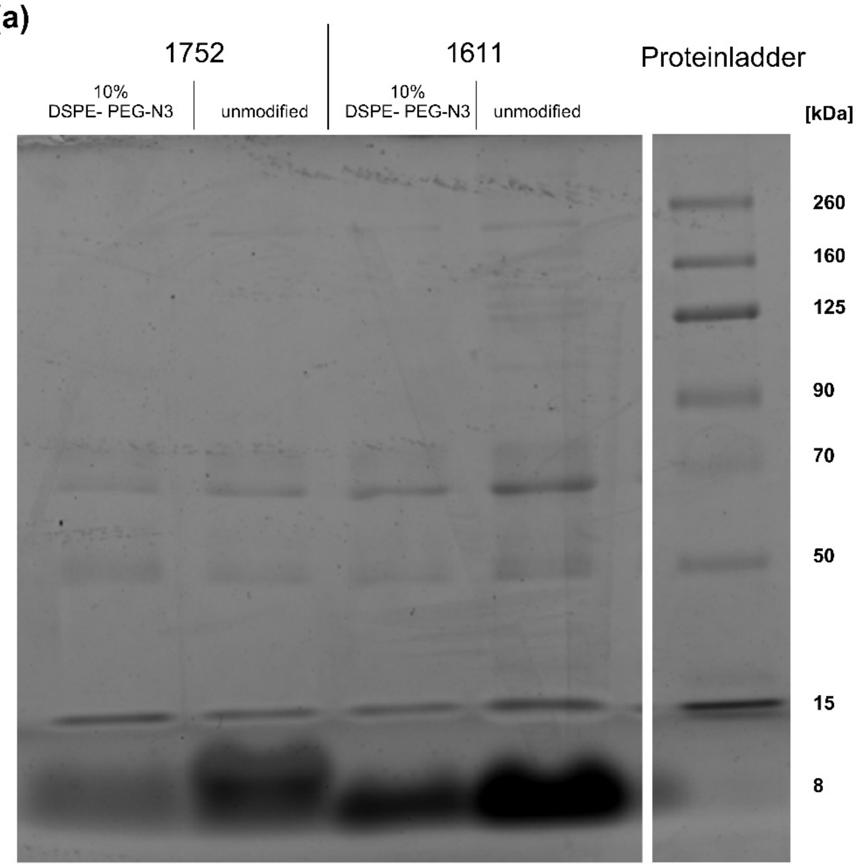

(b)

Unmodified 1611 vs. unmodified 1752

kDa	% Change
70	15.43
15	6.68
8	15.38

Unmodified 1611 vs. 1611 - 10% DSPE-PEG-N_3

kDa	% Change
70	10.33
15	6.38
8	8.87

Unmodified 1752 vs. 1752 - 10% DSPE-PEG-N_3

kDa	% Change
70	3.59
15	-4.46
8	16.36

Figure S10. (a) SDS-PAGE with subsequent coomassie blue staining (imperial protein stain) of mouse serum incubated unmodified and PEGylated (10% DSPE-PEG-N3) LAF-XP mRNA polyplexes. **(b)** Band intensities were quantified using ImageJ. Quantification for the comparison of unmodified 1611 to unmodified 1752 is displayed as percentage change (% change) using the formula $\%\ change = 100 \text{ x} \left(1 - \frac{Intensity\ 1752}{Intensity\ 1611}\right)$ and for the comparison of PEGylated polyplexes against unmodified polyplexes the formula $\%\ change = 100 \text{ x} \left(1 - \frac{Intensity\ PEGylated}{Intensity\ unmodified}\right)$ was used. Three protein bands were quantified at approximately 70, 15 and 8 kDa.

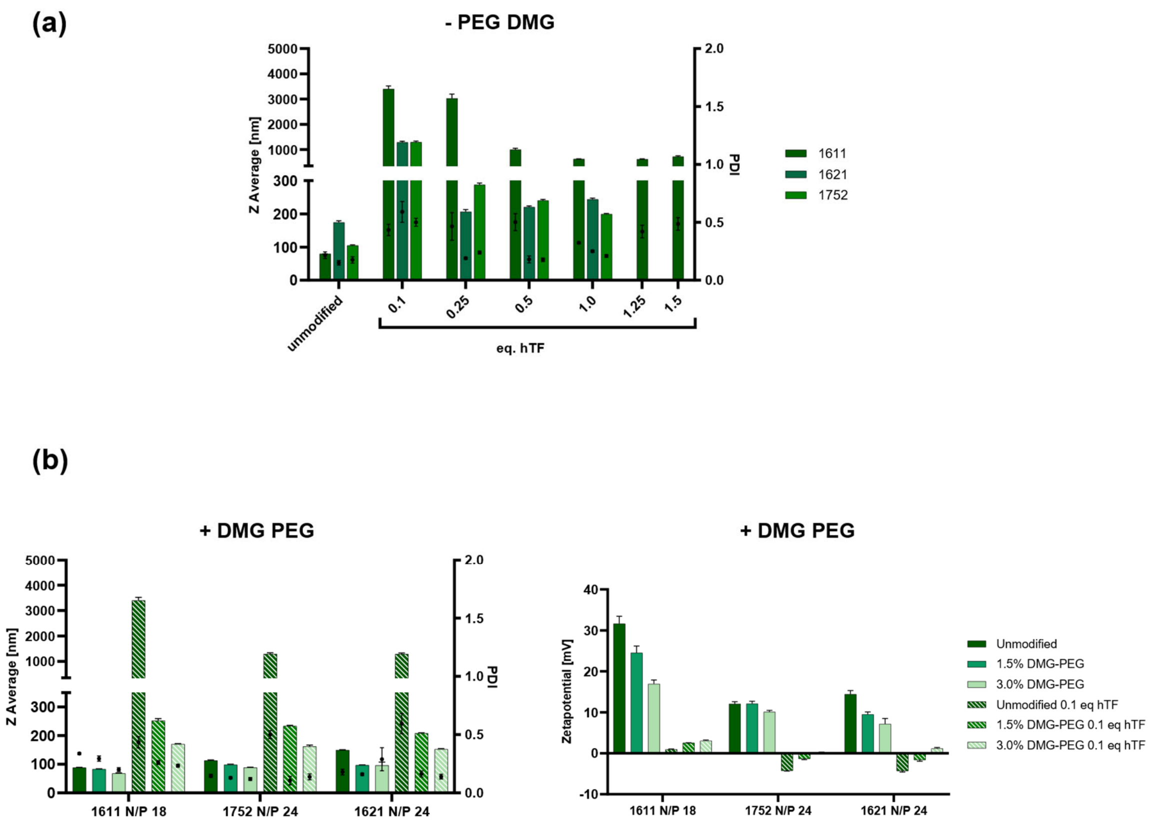

Figure S11. Stabilization of LAF-XP polyplexes against human transferrin (hTF) induced aggregation with DMG-PEG. **(a)** Z-average and PDI of 1611, 1621, and 1752 in the presence of varying molar equivalents (carrier/hTF molar ratio) of human transferrin, measured by DLS. **(b)** Z-average, polydispersity index (PDI), and zeta potential of unmodified and PEGylated (1.5% and 3% DMG-PEG) carriers, both in the presence and absence of 0.1 eq hTF.

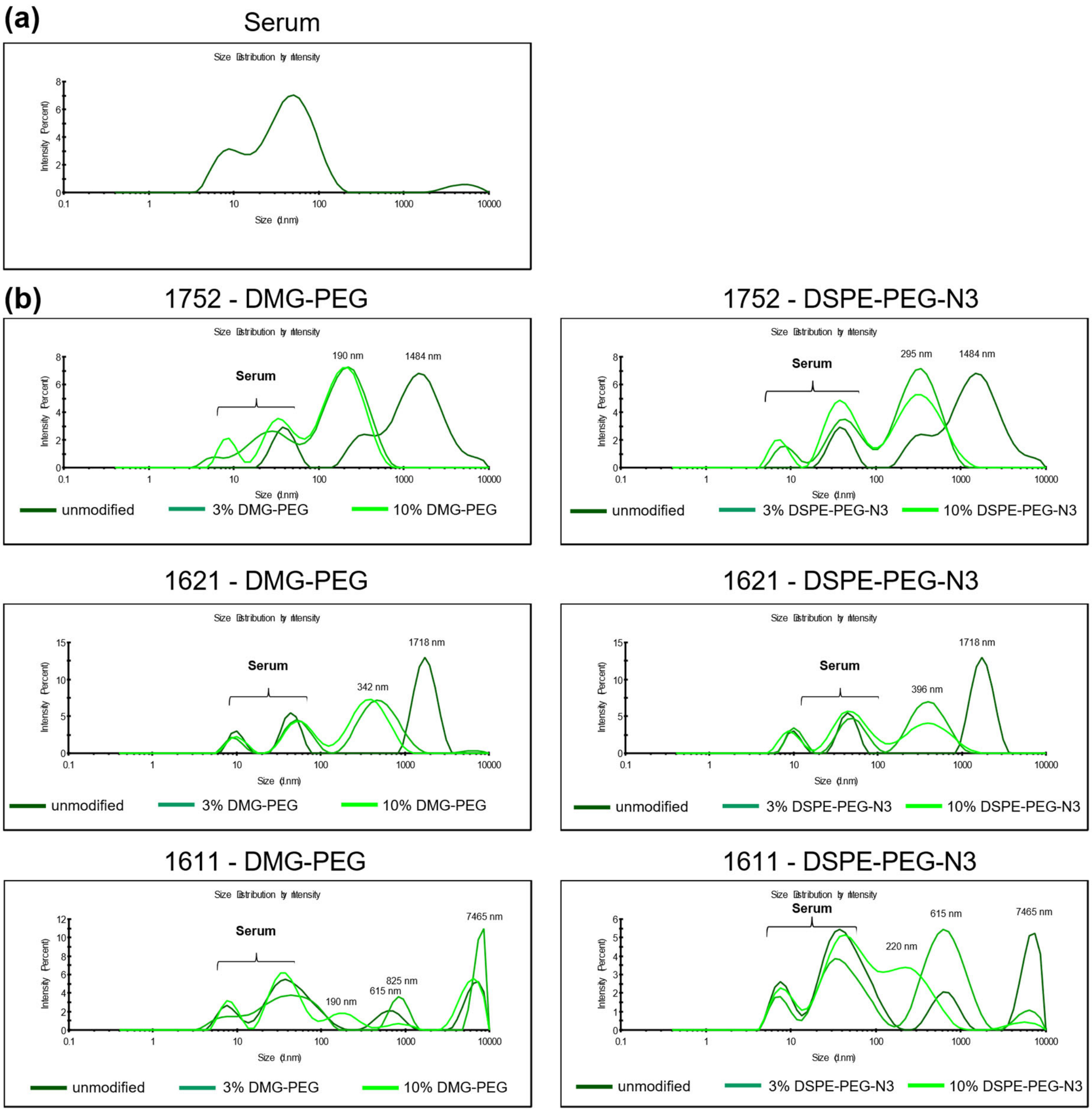

Figure S12. Assessment of particle stability in the presence of serum. Unmodified and PEGylated LAF-XP mRNA polyplexes were diluted and incubated in 90% fetal bovine serum (FBS) for 2 hours at 37°C under continuous shaking at 300 rpm. **(a)** and **(b)** Z average of serum, unmodified and PEGylated LAF-XP polyplexes (3% and 10% DMG-PEG/DSPE) was determined using DLS. For DLS measurements, 40 µL of the FBS-incubated samples were further diluted with 40 µL of HBG, resulting in a final volume of 80 µL, and transferred to a folded capillary cell.

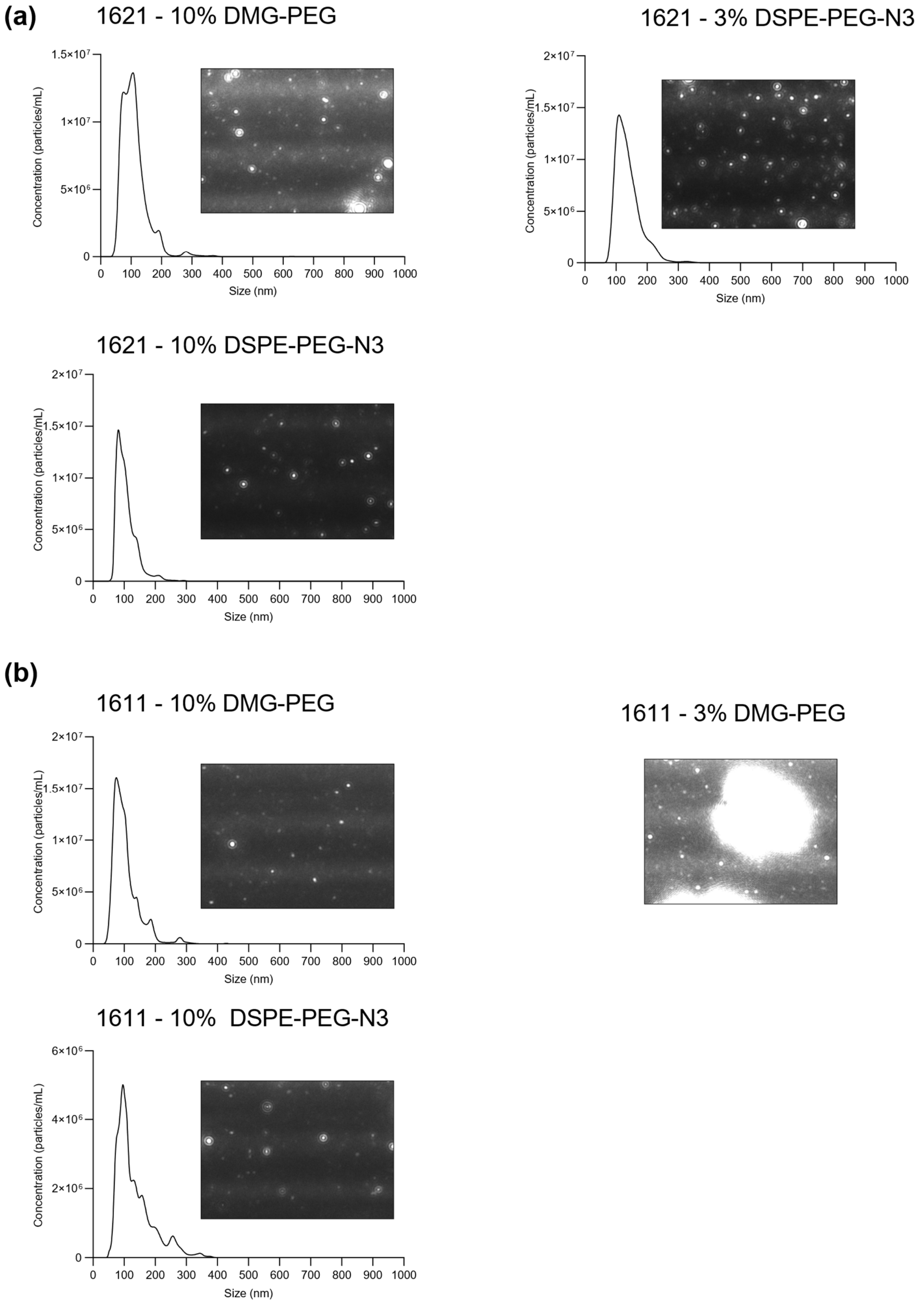

Figure S13. Assessment of stability and functionality in the presence of serum. Unmodified and PEGylated LAF-XP mRNA polyplexes were diluted and incubated in 90% fetal bovine serum (FBS) for 2 hours at 37°C under continuous shaking at 300 rpm. FBS-incubated samples were further diluted 1:200 in Hepes (7.4). **(a)** and **(b)** NTA measurements with the corresponding NTA video frame of unmodified and PEGylated 1621 and 1611 LAF-XP mRNA polyplexes (3% and 10% DMG-PEG/DSPE-PEG-N3).

(a)

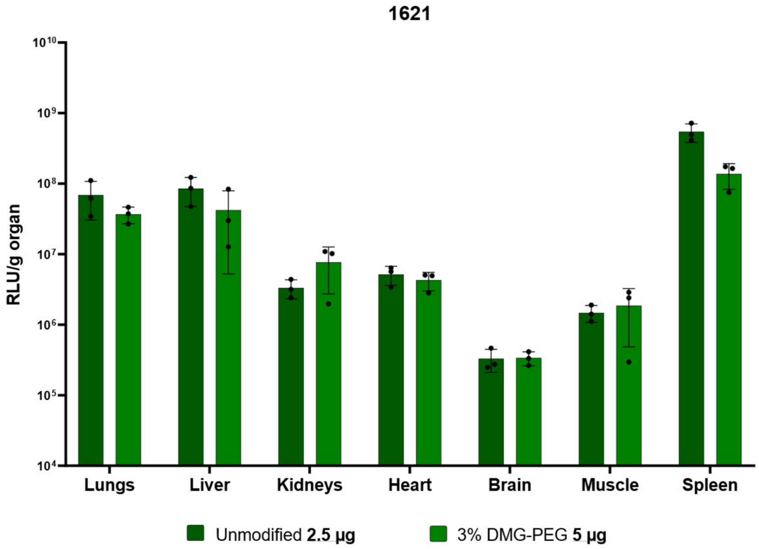

(b)

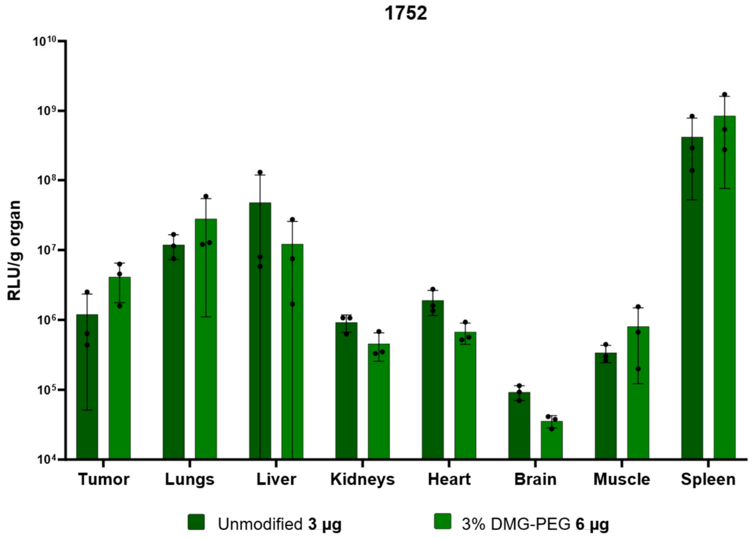

Figure S14. Dose study of 1621 and 1752 LAF-XP mRNA polyplexes. *Ex vivo* luciferase (LUC) assay of organs from A/J mice (1621) **(a)** and N2a tumor-bearing NMRI mice (1752) **(b),** 24 h post-administration (n = 3; mean + SD). Comparison of unmodified 1621 and 1752 polyplexes, both at NP 24, administered at doses of 2.5 µg (1621) and 3 µg (1752), to PEGylated 1621 (NP 24; 3% DMG-PEG) or 1752 (NP 24, 3% DMG-PEG) polyplexes at doses of 5 µg or 6 µg mRNA. Unmodified 1621 NP 24 and 1752 NP 24 at doses of 5 µg (1621) or 6 µg mRNA (1752) caused severe toxicity, resulting in indicated euthanasia shortly after administration.

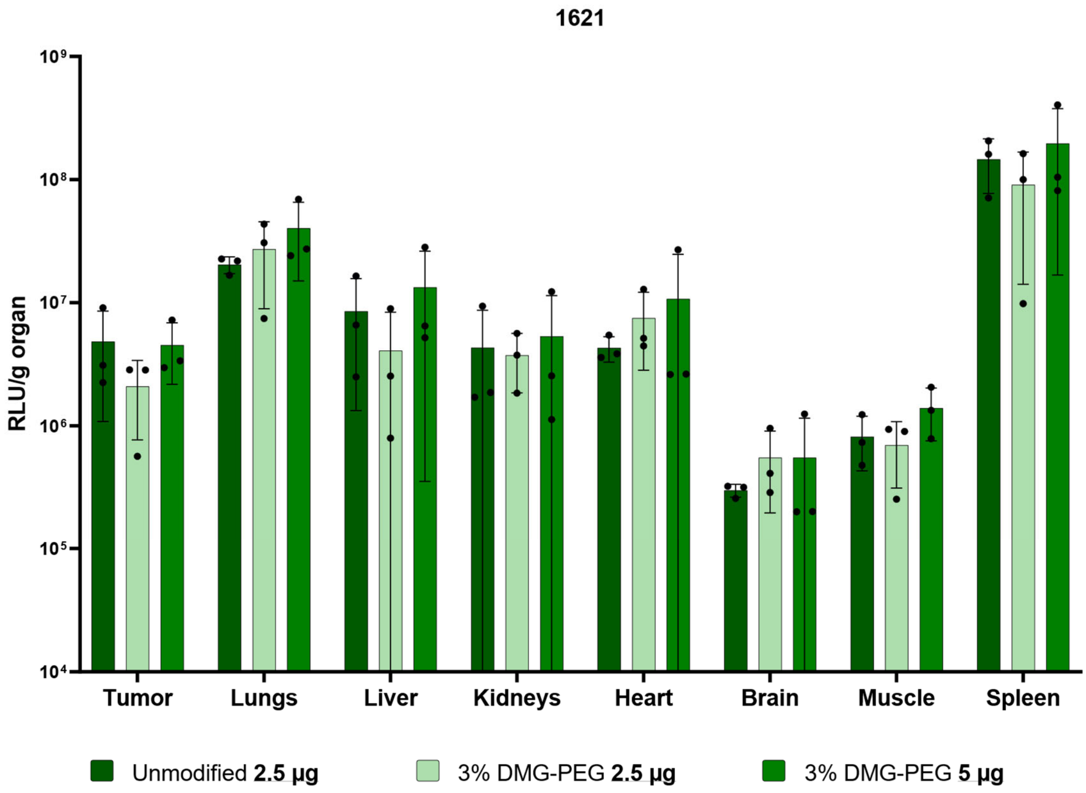

Figure S15. *In vivo* transfection efficiency assessment of unmodified 1621 vs. PEGylated 1621 LAF-XP mRNA polyplexes in N2a tumor-bearing A/J mice. *Ex vivo* luciferase (LUC) assay of organs comparing unmodified 1621 polyplexes at a dose of 2.5 µg mRNA with PEGylated (3% DMG-PEG) 1621 polyplexes at doses of 2.5 µg and 5 µg mRNA, 24 hours post-administration (n = 3; mean ± SD)

S2. Supporting Methods

S2.1. Synthesis of GE11 and scrGE11:

The peptides GE11 (YHWYGYTPQNVI) and scrambled GE11 (scrGE11; YWGPNIHYYTQV) were synthesized using standard Fmoc-based solid-phase peptide synthesis (SPPS) on either isoleucine- or valine-preloaded 2-chlorotityl resin. Synthesis was conducted out on an automated Syro Wave peptide synthesizer (Biotage, Uppsala, Sweden) using Fmoc-protected amino acids and HBTU (2-(1H-Benzotriazol-1-yl)-1,1,3,3-tetramethyluronium-hexafluorophosphat), HOBt (1-hydroxybenzotriazol), and DIPEA (diisopropylethylamine) as standard coupling reagents. Following synthesis, peptides were cleaved from the resin with a mixture of trifluoroacetic acid (TFA), water, and triisopropylsilane (TIS) in a ratio of 95:2.5:2.5 (v/v/v) for 1 hour at room temperature. The cleavage solution was precipitated by dropwise addition into pre-cooled n-hexane/methyl tert-butyl ether (MTBE) (3:1, v/v), followed by centrifugation at 4000 rpm at 4 °C for 15 minutes. The supernatant was discarded, and the resulting pellets was dried under nitrogen stream. For DBCO-functionalization, the crude peptides (15 mg, 0.0096 mmol) were dissolved in 20 mM HEPES buffer and adjusted to pH 8.0-8.5 using 1 M NaOH. DBCO-NHS ester (1.2 eq., 0.0117 mmol, 5.03 mg) was dissolved in anhydrous, degassed DMF and kept on ice for 10 minutes prior to reaction. The DBCO-NHS solutions was combined with the peptide solutions, and the reaction mixture was vortexed briefly. The conjugation reaction was carried out at room temperature for 16 hours. Purification of the peptides was achieved via preparative high-performance liquid chromatography (HPLC) using a LaPrep system (VWR International GmbH,

Darmstadt, Germany) and a SymmetryPrep C18 column (7 µm, 19 x 150 mm). The purified ligands were subsequently lyophilized. The molecular structures were confirmed by matrix-assisted laser desorption/ionization time-of-flight mass spectrometry (MALDI-TOF MS) and analytical reversed-phase HPLC (RP-HPLC)

S2.2. Lyophilization

The synthesized product was lyophilized using a freeze-dryer ALPHA 3-4 LSCbasic (Martin Christ Gefriertrocknungsanlagen GmbH, Osterode am Harz, Germany) with the condenser temperature at -105 °C and a pressure of 0.050 mbar.

S2.3. MALDI-TOF Mass Spectrometry (MS)

MALDI-TOF mass spectrometry was performed using an Autoflex II mass spectrometer (Bruker Daltonics, Germany). A solution of 10 mg mL-1 super-DHB (9/1 (w/w) mixture of 2,5-dihydroxybenzoic acid and 2-hydroxy-5-methoxybenzoic acid) in 69.93/30/0.07 (v/v/v) H_2O/ACN/TFA was used as matrix. 1 µL of matrix solution was added onto a MTP AnchorChip (Bruker Daltonics, Germany). Then, 1 µL of sample dissolved in H_2O/EtOH at a concentration of 1 mg mL-1, was added onto the matrix, co-crystallized and examined. Spectra were recorded in positive ionization mode.

S2.4. Analytical Reversed-Phase HPLC

Reversed-phase high-performance liquid chromatography (RP-HPLC) was performed using a VWR-Hitachi Chromaster 5160 pump system, a VWR-Hitachi Chromaster 5260 autosampler, and a diode array detector (DAD) (VWR-Hitachi Chromaster 5430; VWR, Darmstadt, Germany) with detection at 214, 260, 280 and 308 nm. Separation was performed on a Hydrosphere C18 column (5 µm, 150 x 4.6 mm I.D.; YMC Europe GmbH, Dinslaken, Germany). The mobile phase consisted of ACN/H_2O containing 0.1% TFA, with a linear gradient from 95:5 (v/v) to 0:100 (v/v) over 25 minutes.

S2.5. Preparative HPLC

Preparative high-performance liquid chromatography (prep-HPLC) was carried out using a Büchi Pure C-830 Prep system (BÜCHI Labortechnik GmbH, Essen, Germany) equipped with a SymmetryPrep™ C18 column (7 µm, 19 x 150 mm; Waters, Milford, Massachusetts, USA). Purification was performed with solvent A (100% ACN with 0.1% TFA) and solvent B (100% H2O with 0.1% TFA), applying a gradient from 95% B to 0% B over 30 minutes at a flow rate of 20 mL/min. Eluents were monitored using a UV/vis detector at 254, 265, 290 and 308 nm.

S2.6. Ethidium Bromide (EtBr) Exclusion Assay

Quantification of EtBr fluorescence was performed using a microplate reader (Spectrafluor Plus, Tecan, Switzerland) with an excitation wavelength of λex = 535 nm and an emission wavelength of λem = 590 nm. LAF-polyplexes were prepared as described above (sections 2.2.8) with a total volume of 50 µL at a final pDNA concentration of 10 µg/mL. After an incubation time of 40 min, LAF-polyplexes were diluted with 250 µL of aqueous EtBr solution (c = 0.5 µg/mL). After further incubation for 10 min at RT, 260 µL of each sample was pipetted into a TPP-ft 96-well-plate and fluorescence intensity was measured. For quantification, a standard calibration curve with free pDNA (linear concentration range from 0 to 10 µg/mL) diluted in HBG was used. The amount of free, i.e., non-compacted pDNA was determined based on the calibration of free pDNA and displayed as percentage of EtBr fluorescence in relation to 100% of free pDNA.

S2.7. Agarose Gel Shift Assay

1% (w/v) agarose gel was prepared by microwave-assisted heating of agarose in TBE buffer (18.0 g of tris(hydroxymethyl)aminomethane, 5.5 g of boric acid, 2 mM EDTA at pH 8 in 1 L of H_2O). 1x GelRed

was added to the solution (i.e., 1:1000-dilution of 1000x GelRed stock solution), after the solution cooled down to about 50 °C. The solution was then poured into an electrophoresis chamber and allowed to cool down further to allow gelation. LAF-polyplexes were formulated as described above (sections 2.2.8) with a total volume of 15 μL at a mRNA concentration of 12.5 μg/mL. Following a 40 min incubation, 3 μL of loading dye (6x; prepared from 6 mL of glycerol, 1.2 mL of 0.5 M EDTA, 2.8 mL of H_2O, 0.02 g of bromophenol blue) was added to the LAF-polyplex solution. Subsequently, 15 μL of each sample was loaded to the gel and electrophoresis was conducted at 120 mV for 70 min in 1x TBE buffer. As a control, free mRNA diluted in HBG/H_2O 50/50 to a concentration of 12.5 mg/mL was used.

S2.8. Cell Viability Assay by MTT

Transfections of LAF - polyplexes were performed as described (section 2.2.8). At 24 h post transfection, 10 μL of MTT solution (3-(4,5-dimethylthiazol-2-yl)-2,5-diphenyltetrazolium bromide; 5 mg/mL) was added to each well. Plates were then incubated for further 2 h at 37°C. Subsequently, the supernatant was carefully removed. The plates were stored overnight at -80°C. The resulting purple formazan was then solubilized in 100 μL of DMSO and incubated for 30 min at 37°C with gentle, continuous shaking. Spectophotometric analysis was performed with a Tecan microplate reader (Spectrafluor Plus, Tecan, Männedorf, Switzerland). Absorbance was measured at wavelength λ = 590 nm with a background correction at λ = 630 nm. Relative metabolic activity was related to control well treated with HBG/H_2O (50/50) and calculated by dividing [A]sample/[A]control.

S2.9. Ribo Green Assay

Encapsulation efficiency [ee(%)] of mRNA LAF - polyplexes was evaluating using the Quant-iT™ RiboGreen RNA Assay-Kit (Thermo Fisher Scientific). LAF - polyplex solutions were formed as described above (sections 2.2.8) and subsequently diluted with 1 x TE (10 mM Tris-HCl, 1 mM EDTA, pH 7.5 in RNase-free water) to a concentration of 2 μg/mL. 50 μL aliquotes were either mixed with 50 μL of 1x TE as untreated controls or added to 50 μL of 1x TE containing 2% (v/v) TritonX-100 and 250 I.U. mL-1 heparin to fully dissociated complexes. All samples were incubated at 37 °C for 10 min, while constantly shaking at 150 rpm. Following 5 min cooling period at RT, 100 μL of RiboGreen reagent diluted 200-fold in 1x TE were added to every sample. Then after 5 min, the fluorescence intensity was measured in duplicates using a Tecan microplate reader (Spectrafluor Plus, Tecan, Männderdorf, Switzerland) at excitation/emission wavelength of 485/535 nm. Background signals were measured with pure HBG in 1x TE, or in 1x TE supplemented with TritonX- 100 and heparin, which were treated identically to the respective LAF - polyplex samples. Encapsulation efficiency [ee(%)] was calculated using the following formula after background subtraction of each sample:

$$ee\ (\%) = 100\% - \frac{\text{mean emission untreated control}}{\text{mean emission treated sample}}\ x\ 100\%$$

S2.10. pH Dependent Changes in ZEta Potential of Unmodified and Shielded LAF-XP mRNA Polyplexes

LAF-XP mRNA polyplexes were formulated as described in section 2.2.3, using a concentration of 25 μg/mL at a total volume of 80 μL. The polyplex solution was evenly split into two portions. One portion was diluted with 760 μL of HEPES buffer at pH 7.4 and the other with 760 μL of Hepes buffer at pH 5.4, resulting in a final volume of 800 μL for each sample. After 10 minutes of incubation, size and zeta potential were measured by DLS.

S2.11. In Vivo Performance of mRNA LAF-XP-Polyplexes in Tumor-Bearing Mice:

In vivo experiments were performed according to the guidelines of the German Animal Welfare Act and were approved by the animal experiments ethical committee of the Government of Upper Bavaria (accreditation number Gz. ROB-55.2-2532.Vet_02-19-20). N2a cells (10^6 cells/150 µL PBS) were subcutaneously inoculated into the left flank of 6-week-old female A/Jmice (Envigo RMS GmbH, Düsseldorf, Germany) and NMRI mice (Janvier, Le Genest-Saint-Isle, France). Mice were randomly divided into groups of five and were housed in isolated ventilated cages under specific pathogen-free conditions with a 12 h day/night cycle, and food and water provided ad libitum. Body weight and general well-being were monitored regularly. Tumor size was measured with a caliper and determined by the formula: [0.5 xx (longest diameter) x (shortest diameter)^2]. When tumors reached a size of 250-500 mm^3, the experiments were performed by intravenous tail vein injection of polyplexes formed at indicated N/P ratio, containing 2.5 or 5 µg of mRNA-luc in 150 µL HBG/H_2O (50:50 v/v). An HBG with double the amount of glucose was used to reach isosmotic conditions. Mice were euthanized 24 h after injection. The organs (tumor, lungs, liver, kidneys, spleen, brain, heart, muscle, i.e., hamstring muscles and calves) were carefully dissected and washed with PBS, followed by analysis via *ex vivo* luciferase gene expression assay. The luciferase expression was determined as described below and presented as relative light units per gram organ after background subtraction (lysis buffer).

S2.12. Ex Vivo Luciferase Expression Assay of Organs and Tumors:

Tumor tissues and organs were homogenized in Luciferase Cell Culture Lysis Reagent 1x, supplemented with 1% (v/v) protease and phosphatase inhibitor cocktail with mechanical a tissue and cell homogenizer (FastPrep-24, MP Biomedicals, USA). Subsequently, the samples were stored frozen overnight at −80 °C to ensure full lysis and complete cell disruption. Before measurement, the samples were thawed and centrifuged for 10 min at maximum speed (≈13 000 rpm) and 4 °C. Luciferase activity in 25 µL of the supernatant was determined in a Centro LB 960 plate reader luminometer (Berthold Technologies, Bad Wildbad, Germany) for 10 s after addition of 100 µL/well of a LAR assay buffer solution (composition see above) supplemented with 5% (v/v) of a mixture of 10 mm luciferin and 29 mm glycyl- glycine.
